# Acknowledgment to Reviewers of *Cells* in 2020

**DOI:** 10.3390/cells10020244

**Published:** 2021-01-27

**Authors:** Cells Editorial Office

**Affiliations:** MDPI AG, St. Alban-Anlage 66, 4052 Basel, Switzerland

Peer review is the driving force of journal development, and reviewers are gatekeepers who ensure that *Cells* maintains its standards for the high quality of its published papers. Thanks to the cooperation of our reviewers, in 2020, the median time to first decision was 14.7 days and the median time to publication was 37 days. The editors would like to express their sincere gratitude to the following reviewers for their precious time and dedication, regardless of whether the papers were finally published:

The editors also express their sincere gratitude to all of the following, who shared their time and expert opinion by reviewing for *Cells* in 2020:
Abba, Mohammed L.Liebl, FaithAbd Elmageed, ZakariaLiepins, JanisAbdelalim, Essam M.Liesa, MarcAbdel-Latif, AhmedLightfoot, Adam P.Abdel-Whab, El-SayedLi-Kroeger, DavidAbdi, RezaLiktor-Busa, ErikaAbdul-Muneer, P. M.Lillig, Christopher HorstAbe, YoichiroLim, Dong JunAbken, HinrichLim, Ki-TaekAblashi, DharamLim, RebeccaAbouAlaiwi, WissamLim, Sai-KiangAbramyan, JohnLim, Seung-OeAbsoud, MichaelLim, WonbongAbugri, Daniel A.Lim, Yun-PingAch, ThomasLimagne, EmericAcharya, MunjalLimana, FedericaAcosta-Martinez, MericedesLin, Chang-ShenAdachi, YasushiLin, Chi-HungAdam, Stephen ALin, Dar-ShongAdamek, AgnieszkaLin, GuitingAdameyko, IgorLin, Hsiang-YuAdinolfi, Luigi ElioLin, Huang-YunAdjaye, JamesLin, Muh-ShiAdornetto, AnnagraziaLin, Peter P.Adunyah, Samuel E.Lin, QishanAdur, MalavikaLin, ShengdaAfasizheva, InnaLin, Shih-YiAffourtit, CharlesLin, TaoAfolayan, Adeleye J.Lin, Tsu-KungAgam, GalilaLin, Tzer-BinAgarwal, PoonamLin, WenyuAgarwal, SumitLin, Wey-jinqAgnelli, LucaLin, YaoAgosti, ValterLin, YibinAgrimi, GennaroLin, Yuh-YihAguilar-Carreno, HectorLin, ZhiweiAguirre, AitorLincoln, JoyAhmad, AamirLindahl, LasseAhmad, AliLindahl, Lise M.Ahmad, ShakilLindner, LarsAhmed, Atif AliLindqvist, ArneAi, XiaLindsay, Andrew J.Aicheler, RebeccaLinette, Gerald PAirenne, Tomi T.Link, Christopher D.Akahoshi, MitsuteruLinton, Kenneth J.Akam, Elizabeth CLiobikas, JuliusAkassoglou, KaterinaLipińska, AndreaAkbari, OmidLippmann, EthanAkita, SadanoriLisby, MichaelAkiyama, TasukuLisowska, KatarzynaAktaş, TuğçeLisowska, Katarzyna M.Aktories, KlausList, EdwardAlaimo, AlessandroListrat, AnneAlaimo, SalvatoreLitak, JakubAlam, PerwezLitvinov, Rustem I.Alarcón, BalbinoLitvinova, LarisaAlbano, EmanueleLitwin, Ireneusz BernardAlbano, FrancescoLiu, AiminAlbert, AntonyLiu, BinAlbert, MareikeLiu, Cheng-HsienAlbert, SilviaLiu, Chi-MingAlbi, ElisabettaLiu, Chung-JungAlbrecht, ElkeLiu, DavidAlbrecht, JanLiu, GaowenAlbrecht, RandyLiu, HudanAlcántara-Ortigoza, Miguel AngelLiu, Jun-PingAlcendor, Donald J.Liu, LixinAlché Ramírez, Juan De DiosLiu, MengyangAldea, MartiLiu, Ming-LinAldinucci, DonatellaLiu, MoAlejandre-Alcazar, Miguel A.Liu, Nai-KuiAlessandro, FanzaniLiu, NingAlexaki, Vassilia IsminiLiu, PengdaAlexander, H. DenisLiu, QinAlexandre, MezentsevLiu, Shing-HwaAlferink, JudithLiu, Wei-HsiuAlgar, ElizabethLiu, XiangleiAl-Hasani, HadiLiu, XiangshengAli, Hayssam M.Liu, Yi-ChangAli, HydarLiu, Yi-ShiuanAli, Ibne K. M.Liu, YonggangAlibardi, LorenzoLiu, Yong-YuAl-Khrasani, MahmoudLiu, ZhonghuaAllakhverdiev, SuleymanLivanos, PantelisAllan, AlisonLiverani, ElisabettaAllard, BenoitLizano, MarcelaAllison, LizabethLlobet, ArturAllison, W. TedLlorente, IreneAlloatti, GiuseppeLo, Chunmin C.Alloza, IraideLo, Hui-WenAllweiss, LenaLo, Jeng-FanAlmansa, InmaculadaLobaccaro, Jean-MarcAlmeida, Gabriela MLobanova, Ekaterina S.Almerico, Anna MariaLobo, Glenn P.Al-Mubarak, Bashayer R.Lobo, JoaoAlonso, Miguel A.Locascio, AnnamariaAlonso-Colmenar, Luis M.Lodi, AlessiaAlonso-González, CarolinaLoebel, ClaudiaAlpar, DonatLogarinho, ElsaAlpaugh, KatherineLoirand, GervaiseAlpers, CharlesLolkema, Martijn P.Alpini, GianfrancoLonardo, AmedeoAlsaab, HashemLonati, CaterinaAl-Sawaf, OthmanLončarek, JadrankaAlshareef, AbdulraheemLondei, PaolaAltan-Bonnet, NihalLongley, DanAltman, Brian J.Longo, VitoAltruda, FiorellaLongone, PatriziaAltucci, LuciaLooi, KevinAlvarado-Kristensson, MariaLopalco, GiuseppeAlvarez De La Rosa, DiegoLopatina, TatianaÁlvarez Martín, JavierLopes, Helena BachaAlvarez, Jorge IvánLópez Malo, DanielAlvarez-Rodriguez, ManuelLopez, Jose JavierÁlvarez-Sánchez, NuriaLopez-Camarillo, CesarAlves, C. HenriqueLópez-Camarillo, CésarAlves, Lysangela RLópez-Hernández, Luz BereniceAlves, MarcoLópez-Hernández, YamiléAlves, NunoLópez-Ruiz, ElenaAlvisi, GualtieroLopitz Otsoa, FernandoAly, HusseinLoppin, BenjaminAmadio, PatriziaLopresti, PatriziaAmado, SandraLorenzo González, ÓscarAmagase, KikukoLorico, AurelioAmaral, Joana D.Lortz, StephanAmaral, RonaldoLotti, FrancescoAmato, RosarioLou, Yan-RuAmbrose, ChrisLovat, FrancescaAmbrosio, SantiagoLovatt, MatthewAmente, StLowe, MartinAmes, James BLowery, AoifeAmiel, EyalLoza-Coll, Mariano AndresAmils, RicardoLozano, R. M.Amiral, JeanLu, AlbertAmiya, EisukeLu, FengminAmmazzalorso, AlessandraLu, Hong S.Ammendola, RosarioLu, HongxuAmorim, Maria JoaoLu, HuaAmpofo, EmmanuelLu, Jeng-WeiAmrani, AbdelazizLu, LinchaoAmson, RobertLu, LingengAmthor, HelgeLu, LixiaAn, Hyun JooLu, Mei-ChinAn, PingLu, QingxianAn, SeungLu, QuanlongAna María, Zamora MartínezLu, WeiqinAnand, Paras K.Lu, Wei-YuAnand, RuchikaLu, Yong-ChenAnderluh, MarkoLualdi, MartaAndersen, Ditte C.Lubecka-Pietruszewska, KAndersen, Kasper L.Lucacchini, AntonioAnderson, MicheleLucchetti, DonatellaAnderson, PeterLucchini, FrancoAndersson, UlfLuchetti, AndreaAndjelkovic, Anuska V.Luchini, ClaudioAndó, IstvánLuciani, Giovanni BattistaAndreeva, ElenaLucio, VictorAndreotti, AmyLucyshyn, DorisAndreotti, GiuseppinaLue, Lih-FenAndres, AntonioLuen, StephenAndrés, María T.Lugović-Mihić, LiborijaAndreucci, ElenaLuís, Ana LúciaAndrews, Melissa R.Lukomska, EwaAndrysic, ZdenekLumniczky, KatalinAng, Lay TengLund, Elizabeth K.Angel, Catherine ElizabethLunt, PeterAngeli, AndreaLuo, DahaiAngelidis, CharalamposLuo, LinAngelini, CorradoLuo, XuAngelino, DonatoLuo, Yong-ZhangAngelopoulou, KaterinaLurz, EberhardAngelova, AngelinaLuthra, PriyaAnger, MartinLutz, Sarah E.Angioni, Maria MaddalenaLuxton, G. W. GantAnglani, FrancaLuz-Crawford, PatriciaAngrand, Pierre-OlivierLy, TonyAniello, FrancescoLyck, RuthAniento, FernandoŁyczakowski, JanAnifandis, GeorgeLymperopoulos, AnastasiosAnjos-Afonso, FernandoŁysiak, GrzegorzAnnunziata, Christina M.Ma, LiangAnsari, ShariqMa, RuiAnsorena, EduardoMa, ShuangtaoAntebi, Yaron E.Ma, Wen-JuanAntinozzi, CristinaMa, Zheng FeeiAntipova, VeronicaMaas, CoenAntohe, FeliciaMaass, KendraAntonin, WolframMäättä, Jorma AAntonioli, LucaMacchiarelli, GuidoAntony, Marie LueMacedo, FatimaAoudjit, FawziMacedo, PaulaApolloni, SavinaMacek Jilkova, ZuzanaAppenzellar-Herzog, ChristianMacGowan, Guy AndrewApte, UdayanMachado, IsidroAránega, AmeliaMacín, Stella MarisAraújo, Inês M.Macip, SalvadorAraya, Víctor CarrielMackiewicz, AndrzejArber, CarolineMackowiak, MarzenaArcangeli, Marie-LaureMadak-Erdogan, ZeynepArcella, AntonellaMadaro, LucaArcucci, AlessandroMadduri, SrinivasArellanes-Robledo, JaimeMadeddu, PaoloAretini, PaoloMademtzoglou, DespoinaArevalo, Juan CarlosMader, RobertAricò, EleonoraMader, SylvieArif, TasleemMadka, VenkateshwarArii, JunMadrid, MarisaArikawa-Hirasawa, EriMadry, MagaliAriyani, WindaMadsen, Daniel HargbølArlt, AlexanderMadureira, Patrícia A.Armesilla, AngelMaekawa, MasamitsuArmstrong, MichelleMaekawa, RyoAroca, AngelesMaekawa, ShinyaArranz, AmaiaMaeng, Han-JooArteel, Gavin E.Maggi, Leonard B.Arthur, ChrisMaggiolini, MarcelloArtner, IsabellaMagi, SimonaArts, Rob J.W.Magnaghi, ValerioArufe, María CMagnan, BrunoArumugam, GarrieMagni, PaoloAsahara, TakayukiMagnusson, Karl-EricAsante, EmmanuelMagnusson, PerAscencio-Ibanez, TrinoMagrané, JordiAsgari, NasrinMagrassi, LorenzoAsgharpour, AmonMahjoub, Moe R.Ashihara, EishiMai, SabineAskjaer, PeterMaia, CláudioAspenström, PontusMaiato, HelderAssinck, PeggyMailloux, RyanAster, Jon CMains, Richard E.Aster, Jon C.Maione, SabatinoAstori, GiuseppeMaisano, DomenicoAszodi, AttilaMaity, ShuvadeepAtanackovic, DjordjeMaiuri, PaoloAtawia, Reem T.Majello, BarbaraAthanasiou, Labrini V.Majhen, DragomiraAthauda, DilanMajima, Hideyuki J.Atkin, StephenMajor, BenAtochin, DmitriyMajumder, AvisekAtwood, Scott X.Majumder, MrinmoyeeAtze, BergsmaMakarevich, PavelAuburger, GeorgMakiyama, TakeruAudrito, ValentinaMalaguarnera, GiuliaAugust, AveryMalapelle, UmbertoAugustyniak, DariaMalashicheva, AnnaAuladell, CarmeMalavasi, FabioAurora, RajeevMalavolta, MarcoAurrand-Lions, MichelMalbon, AlexandraAutry, JosephMalejczyk, JacekAu-Yeung, Chilam AuMalemud, CharlesAvazmohammadi, RezaMalemud, Charles J.Avignone, ElenaMalfitano, Anna MariaÁvila, JulioMalik, Anna R.Avni, OrlyMalinsky, JanAvoli, MassimoMalkovsky, MiroslavAvramenko, Tatiana V.Mallis, PanagiotisAvramidou, EvangeliaMalpeli, GiorgioAvtanski, DimiterMalwal, Satish R.Awate, SanketMalý, PetrAxtell, RobertMamidi, NarmidiAyon, RamonMammadova-Bach, ElminaAzad, ArunMammoto, AkikoAzer, Samy A.Mancini, RitaAzimzadeh, JulietteManda-Handzlik, AnetaAzimzadeh, OmidMandard, StéphaneAzizi, EbrahimMandatori, DomitillaAzuma, YasutakaManea, AdrianBaaße, AnnemarieManfredi, FrancescoBaba, YuhManfredi, InAngeloBacci, StefanoMani, SridharBacete, LauraManickam, RavikumarBach, LeonManilay, Jennifer O.Bacher, UlrikeManna, IdaBachmann-Gagescu, RuxandraManna, SaikatBadea, Tudor ConstantinMannell, HannaBadens, CatherineMänner, JörgBadimón, LinaManni, SabrinaBadr, ChristianMannion, ArleneBae, Chang-HyuManoury, Beńed́icteBae, SangsuMansoor, AdnanBaer, MarkusMansuri, ShahidBaer, Patrick C.Mantovani, RobertoBaez-Nieto, DavidMantripragada, Venkata P.Baggi, FulvioMantuano, ElideBaglietto-Vargas, DavidManzo, EmilianoBagnoli, PaolaManzotti, GloriaBagyinszky, EvaMao, XumingBaharom, FaezzahMarasco, DanielaBai, BingMarazuela, MonicaBailey, Charles GMarazziti, DanielaBailey, Shannon M.Marc, DanielBailly, ChristianMarcal, HelderBaj, AndreinaMarceau, FrançoisBaj-krzyworzeka, MonikaMarchese, CinziaBak, Lasse K.Marchese, MariaBaker, Mark D.Marchetti, AlessandraBakkali, MohammedMarchetti, PhilippeBaklouti, FaouziMarchetti, SandrineBakowska-Zywicka, KamillaMarchuk, Douglas A.Balaban, Amanda E.Marcon, LucianoBalajthy, ZoltánMardešić-Brakus, SnježanaBalatsos, NikolaosMarechal-Drouard, LaurenceBalbi, CarolinaMarei, HanyBalbir-Gurman, AlexandraMareschi, KatiaBaldo, GuilhermeMarfany, GemmaBaldovino, SimoneMarfurt, JuttaBalduini, WalterMargadant, CoertBalestra, DarioMargaritis, LukasBalestrini, Raffaella MariaMargheri, FrancescaBalla, AndrásMarginǎ, DenisaBalla, JozsefMargis, RogerioBalmer, LoisMaría Rosaura, Leis TrabazoBalogi, ZsoltMarian, BrigitteBalzan, SilvanaMarian, CatalinBalzano, TizianoMariani-Costantini, RenatoBamford, ConnorMarigo, ValeriaBanaszynski, Laura A.Marimpietri, DaniloBanciu, ManuelaMarini, MarinaBandapalli, Obul ReddyMarini, Patricia EstelaBandopadhyay, RinaMarino, FrancaBandyopadhyay, BidhanMariot, VirginieBanerjee, AditiMariotti, CaterinaBanerjee, DebabrataMariussen, EspenBanerjee, Hirendra NathMarkiewicz, RoksanaBanfalvi, ZsofiaMarkopoulos, AnastasiosBang, SangsuMaroni, PaolaBangi, ErdemMaroteaux, LucBank, IlanMarques, TaniaBanks, Alexander S.Marracci, SilviaBano, DanieleMarrelli, MassimoBapputty, ReenaMarrs, James A.Bär, SéverineMarsano, René MassimilianoBaraban, JayMarsche, GuntherBarabas, PeterMarsh, Leigh M.Barakat, Tahsin StefanMartensen, Pia MBaran, JaroslawMarteyn, AntoineBarančík, MiroslavMartí, JoaquínBarauskas, GiedriusMartikainen, MiikaBarbalho, Sandra MariaMartikainen, RiikkaBarber, AmoretteMartín Hernández, RobertoBarbero, AndreaMartin, Elizabeth M.Barbieri, ElenaMartin, FranciscoBarbon, AlessandroMartin, FranckBarbon, SilviaMartin, JacintaBarboni, BarbaraMartin, Luc J.Barceló-Coblijn, GwendolynMartín, MargaritaBard, FredericMartin, PatriciaBardag-Gorce, FawziaMartin, SallyBareja, AkshayMartin, Sophie G.Bari, EliaMartín-Antonio, BeatrizBarichello, TatianaMartín-Aragón, SagrarioBarilani, MarioMartinat, CécileBarile, LucioMartín-Cordero, LeticiaBarille-Nion, SohieMartín-de-Llano, José JavierBarna, JánosMartinelli, GiovanniBarnea, Eytan RMartínez Contreras, Rebeca DeboraBarnes, Betsy J.Martínez De Pablos, RocíoBarone, EugenioMartinez, José LuisBarragán, IsabelMartinez, MercedesBarresi, VincenzaMartínez-Chantar, María LuzBarreto, GoncaloMartínez-Martínez, IreneBarreto-Chaves, Maria Luisa MoraisMartinho, OlgaBarrett, Robert J.Martini, VeronicaBarrientos, AntonioMartin-Lopez, EduardoBarros-Barbosa, Aurora RaquelMartino, SabataBarteková, MonikaMartinotti, SimonaBartels, DorotheaMartins Lima, AugustoBartelt, AlexanderMartins, IanBarth, BrianMartins, NataliaBarth, RolfMartín-Satué, MireiaBarton, Matthew JMartins-Bessa, AnaBartoszewski, RafalMartín-Vasallo, PabloBaschant, UlrikeMarty, SergeBashford-Rogers, RachaelMarucci, GabriellaBasile, UmbertoMaruthamuthu, VenkatBasini, GiuseppinaMarverti, GaetanoBaskaran, RathinasamyMarx, SébastienBasso, DanielaMarx-Stoelting, PhilipBasso, LilianMarzban, GorjiBasu, DipanjanMarzetti, EmanueleBasu, UttiyaMarzilli, MarioBathula, Chandra SekharMarzo, IsabelBatie, MichaelMarzocco, StefaniaBatissoco, Ana C.Marzoli, FrancescaBattaglia, RosaliaMasahiro, SatoBattiato, AlfioMasalova, Olga V.Battino, MaurizioMascitti, MarcoBattistelli, MichelaMasel, Eva KatharinaBauer-Kreisel, PetraMason, William S.Bavari, SinaMasoumi, ZahraBawage, SwapnilMasquida, BenoitBay, PeterMass, ElviraBayrak, Ömer FarukMassaad, CharbelBayry, JagadeeshMassadi, Omar AlBazán, EulaliaMassie, AnnBazil, JasonMassoudi, DawiyatBear, ChristineMaster, Christina L.Beatson, Richard E.Mastino, AntonioBeaujean, NathalieMastropasqua, LeonardoBebbere, DanielaMasutani, MitsukoBechstedt, SusanneMasyuk, AnatoliyBeck, William T.Mata, AnaBecker, ThereseMatallanas, DavidBednarek, PiotrMathe, EwyBeer, Hans-DietmarMather, Karen A.Beghini, AlessandroMátis, GáborBeharry, Kay D.Matoba, KeiichiroBehbahani, HomiraMatouskova, PetraBehling, FelixMatozaki, TakashiBehrends, ChristianMatsubara, ShigekiBehringer, ErikMatsui, ReikoBehzad, MansooriMatsumoto, TaroBeitz, EricMatsumura, TsuyoshiBekri, SoumeyaMatsuo, MasafumiBelaidi, Abdel AliMatsuya, YusukeBelfiore, AntoninoMatsuzaki, ToshiyukiBelgiovine, CristinaMatta, CsabaBeliakova-Bethell, NadejdaMattei, BenedettaBelicchi, MarziaMattei, VincenzoBellan, MattiaMatter, KarlBellavia, DanieleMatthews, ShawanaBellelli, RobertoMatucci Cerinic, MarcoBellezza, IlariaMatulić, MajaBello, MartinianoMatusali, GiuliaBelluzzi, ElisaMatuschewski, KaiBelosludtsev, KonstantinMatveevsky, SergeyBelsham, Denise D.Maugeri, Andrea GiuseppeBel’skaya, LyudmilaMauk, Michael G.Beltrami, Antonio PaoloMaurer, Sarah E.Benabdellah, KarimMaurizio, MartiniBen-Ari, YehezkelMauro, AnnunziataBendahhou, SaïdMavangira, VengaiBendas, GerdMavilio, DomenicoBen-Dov, Iddo Z.May, Lauren T.Benedetti, AntonioMayer, AndreasBen-Hur, AsaMayor Jr., FedericoBenitez, SoniaMayor Ruiz, CristinaBen-Nissan, GiliMay-Simera, HelenBenson, D.l.Mazurov, Dmitriy VBentrop, JoachimMazzucato, MarioBen-Ze’ev, AvriMbalaviele, GabrielBerardo, ClarissaMcArthur, Jeffrey RBerce, CristianMcCaffery, PeterBerdoukas, VasiliosMcCain, Megan L.Beretta, Giovanni L.McCaughan, Geoffrey WilliamBerezhnaya, ElenaMcCormick, JamesBerezin, AlexanderMcculloch, ChristopherBergdahl, AndreasMccully, Kilmer S.Berge, Derk TenMcDaneld, Tara G.Berger, Joseph R.McDermott, SuzanneBerger, MassimoMcdonald, CourtneyBerger, WalterMcEnaney, RyanBerghe, Pieter VandenMcFarlane, Isabel M.Bergmann, AndreasMcGill, JodiBergmann, OlafMcGowan, Stephen E.Berishvili, EkaterineMcGrew, MikeBernacchi, SerenaMcIntosh, J. Richard (Dick)Bernal-Lopez, M. RosaMcIntyre, Jeremy C.Bernard, AmélieMcKay, BrianBernardes De Jesus, BrunoMckenzie, MathewBernardi, SimonaMcMillin, MatthewBernardini, GiuliaMcVey, MitchBernardino, Raquel L.McVicar, Daniel W.Bernatchez, Jean A.Meares, Gordon P.Berndt, Michael C.Mears, JasonBernheim-Groswasser, AnneMeccariello, RosariaBertacchini, JessikaMechtcheriakova, DianaBerthelot, Jean-MarieMeckes, DavidBerti, LuciaMedalia, OhadBertke, AndreaMedas, FabioBertolin, GiuliaMedina, Reinhold J.Bertozzi, GiuseppeMedlock, AmyBertrand, Anne T.Medveczky, PeterBeug, ShawnMeehan, KatieBeutner, GiselaMeerang, MayuraBewick, GuyMeerson, AriBeyer, Eric C.Mees, MaartenBezard, ErwanMège, René-MarcBhagirath, DivyaMego, MichalBhardwaj, RajeshMegraw, Timothy L.Bhargava, AditiMegyeri, KlaraBhat, KamakotiMehlmann, LisaBhati, Kaushal KumarMehrotra, ShikharBhattacharya, KaushikMehta, AnandBhattacharya, SupriyoMehta, Gaurav A.Bhowmick, SauravMehta, GautamBhullar, RajMei, Kuo-ChingBhutia, Yangzom DomaMei, YangBi, XinMeinke, PeterBiagini, GiuseppeMeiser, JohannesBiałek, MałgorzataMeissner, AnjaBianchi Filho, CesarioMeitzler, Jennifer L.Bianchi, VittorioMeka, Durga PraveenBianchini, FrancescaMekori, Yoseph A.Biburger, MarkusMelanitou, EvieBichet, Daniel G.Meleady, PaulaBicker, AnneMelendez-Zajgla, JorgeBido, SimoneMelero, IgnacioBidoli, EttoreMelero, José María MateosBiella, Gerardo RosarioMeli, AlbanoBielska, EwaMelillo, Rosa MarinaBierhoff, HolgerMellor, HerryBierle, CraigMelnyk, Roman A.Biersack, BernhardMelo, RossanaBiffi, GiuliaMelone, Mariarosa Anna BeatriceBigiani, AlbertinoMelton, Douglas ABijlsma, Maarten F.Mendes, Alexandrina FerreiraBilban, MartinMéndez Bolaina, EnriqueBilbao, DanielMendez, AaronBillerbeck, EvaMendez-Otero, RosáliaBilodeau, MarcMenendez, DanielBinarová, PavlaMénétrier-Caux, ChristineBinshtok, Alexander M.Meng, GuangxunBird, Alexander W.Meng, JinhongBird, DavidMennerich, DanielaBirge, Raymond B.Menter, ThomasBirner-Gruenberger, RuthMenzorov, Aleksei GBironaité, DaivaMeraviglia, SerenaBirukov, Konstantin G.Mercatali, LauraBiscetti, FedericoMercier, RaphaelBisoffi, ZenoMerdes, AndreasBisti, SilviaMereiter, StefanBiswas, RoopaMereu, ElisabettaBitter, WilbertMerigo, FlaviaBjorndahl, LarsMerindol, NatachaBjornsson, HansMerino Peláez, GraciaBlaauw, BertMerino, Jose JoaquinBlache, PhilippeMerino, RamónBlack, Bryan JamesMerlo, Giorgio RobertoBlack, Homer S.Merrick, B. AlexBlackwell, T. KeithMerriman, TonyBlair, Harry C.Mesci, PinarBlais, AnneMesirca, PietroBlanch, Richard J.Messa, PiergiorgioBlandino, GiovanniMessori, LuigiBlankesteijn, MatthijsMestroni, LuisaBlanquet, VéroniqueMet, ÖzcanBlaser, MarkMetzinger, LaurentBlas-Garciá, AnaMeyer, ChristianBlasiak, JanuszMi, LanBlaya, DeliaMia, SobujBlazek, DaliborMiano, Maria GiuseppinaBlázquez-Castro, AlfonsoMicha, DimitraBlesson, Chellakkan SelvanesanMichael, VeitBlind, RaymondMichaelis, MartinBliven, Spencer E.Michaelis, SusanBloise, EnrricoMichaeu, OlivierBlum, ArnonMichalek, KatarzynaBlumer, MichaelMichalke, BernhardBłyszczuk, PrzemysławMichaud, MorganeBoard, MaryMichelacci, Yara M.Boaz, KarenMichelhaugh, Sharon KayBobek, VladimirMichetti, FabrizioBobis-Wozowicz, SylwiaMichon, FredericBoccellino, MariarosariaMiekus, KatarzynaBocchi, Edimar AlcidesMier, PabloBockenhauer, DetlefMiere, DoinaBoda, FranciscMierzejewska, JolantaBode, Ann MMiggiano, RiccardoBodea, Liviu-GabrielMigliaccio, AntimoBodin, JohannaMihara, KeichiroBödör, CsabaMijailovich, SrbaBoeckel, Jes-NielsMika, DelphineBoehm, StefanMikala, GaborBoehme, Karen A.Mikelis, ConstantinosBoekema, BoukeMiki, YasuhiroBoeldt, Derek SMikropoulos, ChristosBoesch, MaximilianMikula-Pietrasik, JustynaBoeuf, HélèneMilan, GabriellaBogdanov, Alexey A.Milane, LaraBogie, Jeroen F.J.Milavetz, BarryBogusławska, JoannaMilenkovic, Vladimir M.Bohn, ErwinMilevskiy, Michael J. G.Boirivant, MonicaMiljković, Miloš D.Boitano, ScottMill, Jose GeraldoBollini, SvevaMillay, Douglas P.Bolognini, DanieleMiller, KarlBölükbas, DenizMills, KenBombardi, CristianoMilosavljevic, NinaBommer, GuidoMimmi, SelenaBonaccorso, StefaniaMinafra, LuigiBonam, Srinivasa ReddyMinamisawa, SusumuBonanno, ElenaMinamoto, ToshinariBonato, V.L.Minarovits, JanosBonaventura, AldoMinato, Ken-ichiroBond, Heather M.Minelli, RosalbaBonetti, AlessandroMinguela, AlfredoBönig, HalvardMinic, ZeljkaBonilha, VeraMiosge, NicolaiBonior, JoannaMiotto, BenoitBonner, CarolineMirabelli, CarmenBonni, ShirinMiragoli, MicheleBoomer, Jonathan S.Miranda, EnriqueBooth, BrianMiranda-Gonçalves, VeraBorde, ValérieMiro, JordiBordin, LucianaMirzayans, RazmikBordonaro, MichaelMisaka, TomofumiBorgese, NicaMiserey-Lenkei, StéphanieBorggrefe, TilmanMishra, RosalinBorghini, AndreaMishra, Santosh K.Boris-Lawrie, KathleenMisiak, BłazejBork, KayaMisteli, TomBorlongan, CesarMiszczyk, JustynaBorn, WilliMitchell, Brian JBorovac, Josip A.Mitchell, Cassie S.Borrás, FrancescMitchell, DianaBorska, SylwiaMitchell, John S.Borst, JannieMitkin, Nikita A.Bort, RoqueMitola, StefaniaBortolussi, SilvaMitra, KatsuriBose, TanimaMitra, RamkrishnaBosland, Maarten C.Mitrovic, Milana S.Bosmann, MarkusMitsios, AlexandrosBostjancic, EmanuelaMitsuaki, MoriyamaBôta, AttilaMittal, SonamBoto, TamaraMitton, KennethBott, AlexMiura, NaokiBottani, EmanuelaMiura, NatsukoBoudon, JulienMivechi, Nahid F.Boudou, ThomasMiyagoe-Suzuki, YukoBoudreau, FrançoisMiyata, YasuyoshiBougea, AnastasiaMiyata, YoshihikoBougen-Zhukov, NicolaMiyatake, Shin-IchiBouhassira, EricMiyazaki, KoujiBouloumié, AnneMiyazawa, KeisukeBoulton, Michael E.Miyoshi, DaisukeBouma, Gerrit JerryMizushima, NoboruBourboulia, DimitraMladenov, EmilBourgeois, PatriceMlejnek, PetrBourgoin, SylvainMo, ChenglinBourke, EmerMoccia, FrancescoBouter, AnthonyMoccia, MarcelloBouvard, DanielMock, BeverlyBovin, NicolaiMody, VICKYBowen, Nathan J.Mogilner, AlexBowerman, MelissaMohamed, BashirBoyman, OnurMohamed, RiyazBozidis, PetrosMohammad, Ramzi M.Bozzi, YuriMohd-Yasin, FaisalBracchi-Ricard, ValerieMohr, AndreaBracci, MassimoMOK, Chee KengBracco, EnricoMoktefi, AnissaBracht, ThiloMolares-Vila, AlbertoBrader, GünterMoles, Jean-pierreBradford, BarryMolina-Cerrillo, JavierBradshaw, NicholasMolina-Heredia, Fernando P.Brady, GarethMoll, GuidoBragança, JoséMollace, VincenzoBraicu, CorneliaMollapour, MehdiBrakebusch, CordMøller, Holger JonBrambilla, RobertaMolloy, Peter E.Branch, Matthew JamesMolnár, ElekBranchini, AlessioMonaghan, MichaelBrancolini, ClaudioMondini, MicheleBrand-Saberi, BeateMondino, AnnaBrandt, RolandMonget, PhilippeBranlant, ChristianeMongiat, MaurizioBräuer, Anja UrsulaMongin, Alexander A.Brault, Jeffrey J.Monif, MasturaBraun, RalfMonje, Paula V.Braun, ThorstenMonk, Pete N.Bréchard, SabrinaMonsivais, DianaBrechbuhl, HeatherMontanari, ElitaBregadze, VladimirMontanaro, LorenzoBrehm, RalphMontenarh, MathiasBreier, GeorgMontesinos, M. CarmenBremer, EdwinMonti, EugenioBrennan, EoinMontiel-Duarte, CristinaBrennan, MeadhbhMontone, Kathleen T.Brenner, J. ChadMontpetit, BenBresciani, ElenaMonzón, MartaBresson-Bepoldin, LaurenceMoon, Il SooBrewin, NickMoon, YuseokBria, EmilioMoonen, Carolyn G.J.Briata, PaolaMoore, StuartBriedis, VitalisMoore, Thomas FBrighton, Paul JMoorhouse, AndrewBrinchmann, Monica F.Morais De Sá, EuricoBrini, MarisaMorales, JulioBrinton, MargoMorales-González, José AntonioBrizzi, Maria FeliceMorales-Quintana, LuisBrocca, StefaniaMoreau, ViolaineBrockerhoff, Susan E.Moreira, Ana CarolinaBrodehl, AndreasMoreira-Filho, Carlos AlbertoBroering, RuthMorelli, Maria BeatriceBrone, BertMorello, LauraBronisz, AgnieszkaMorena, FrancescoBronowska, AgnieszkaMoreno, JordiBronte, FabrizioMoreno, SandraBrookes, MathewMoreno-Vinasco, LilianaBrooks, Andrew J.Moresi, VivianaBrooks, Philip JohnMoretti, MartaBros, MatthiasMoretti, MatteoBrotto, MarcoMorgan, ClaireBrouillet, EmmanuelMorgan, EthanBrouwers, Jos F.Morgan, Ethan L.Brown, CarolynMorgan, Joshua TBrown, DennisMorgan, MichaelBrown, PeterMorgese, Maria GraziaBrozovic, AnamariaMorikawa, SatoruBrozyna, AnnaMorimoto, TatsuyaBru, SamuelMorin, NathalieBrucoli, MatteoMoriscot, Anselmo SigariBruder Do Nascimento, ThiagoMorishita, HideakiBrueggen, Marie-CharlotteMorita, MasahiroBrumeanu, Teodor-DoruMorita, MasamuneBrune, WolframMorita, NaokiBrunet De La Grange, PhilippeMoritz, OrsonBruning-Richardson, AnkeMoriyama, TakayaBrünnert, DanielaMorizane, RyujiBruno, AntoninoMorlando, MariangelaBruns, DieterMorleo, ManuelaBrustovetsky, NickolayMoro, AlessandroBryniarski, KrzysztofMoro, LoredanaBrzóska, EdytaMorotomi, TakahikoBucchi, CristinaMorouço, PedroBucci, CeciliaMorozova, OlgaBuchet, ReneMorpurgo, MargheritaBuchheim, JudithMorreau, HansBuck, JochenMorrione, AndreaBuck-Koehntop, Bethany A.Morris, Brian J.Buday, LaszloMorrow, John P.Bueno-Silva, BrunoMorselli, MarcoBufalieri, FrancescaMorser, JohnBuffo, AnnalisaMoschetta, MicheleBukrinsky, MichaelMoschou, PanagiotisBulatov, EmilMoschovi, MariaBulfone-Paus, SilviaMoseley, JamesBullon, PedroMoser, SimoneBultynk, GeertMoshaverinia, AlirezaBunch, HeeyounMosienko, ValentinaBungert, JörgMosqueira, DiogoBunse, LukasMosqueira, MatiasBuolamwini, JohnMoss, BernardBuraschi, SimoneMotakis, EfthymiosBurchell, JoyMotta, Chiara MariaBurch-Smith, TessaMottes, MonicaBurdak-Rothkamm, SusanneMou, LishaBurga, RachelMou, YongchaoBurger, DylanMounier, CatherineBurger, Michael CMountzios, GiannisBurgess, David R.Moura, Marcelo TigreBurk, OliverMoura, RuteBurnett, Barrington G.Mousa, Shaaban A.Burrello, JacopoMousley, CarlBurstyn-Cohen, TalMoussay, EtienneBurton, Zachary FMoutin, Marie-Jo Burvenich, IngridMowat, Freya MBusch, AlbertMozos, IoanaBuschbeck, MarcusMu, Ting-WeiBustos, MatildeMuchir, AntoineButcher, AdrianMucignat, CarlaButler-browne, GillianMuhammad, NaoshadByrne, Frances L.Muir, AlexanderBystrom, JonasMujtaba, ShirazByzova, Tatiana V.Mukaida, NaofumiCaballero-Garcia, BeatrizMukherjee, AmitavaCaballo, Mar CarriónMukhopadhyay, SubhradipCabiati, ManuelaMukhopadhyay, SumanCabral, HoracioMuldoon, Timothy J.Caccamo, DanielaMülleder, MichaelCacciottolo, MafaldaMüller, Hans-Arno J.Caceres, SaraMüller, JanisCacialli, PietroMüller, LudmilaCadamuro, MassimilianoMuller, MarcCadenas, SusanaMuller, MiryamCadilla, CarmenMuller, Patricia A. J.Caetano, GuilhermeMullineaux, PhilCafforio, PaolaMullins, Robert FCagnin, StefanoMullins, Robert F.Cahill, MichaelMullins, Roger J.Cai, GiampieroMulthoff, GabrieleCai, HoujianMuniraj, NethajiCai, JasonMuñoz, Estela M.Cai, LishengMuñoz-Galván, SandraCai, YingyunMuntane, JordiCaiazzo, ElisabettaMunusamy, ShankarCaiazzo, MassimilianoMuraca, MaurizioCaignard, AnneMurakami, KosakuCairrão, ElisaMurakami, YasufumiCakstina, IneseMurakami, YoshikiCalabrese, FiorellaMuraki, KatsuhikoCalado, Cecília R.C.Muranov, KonstantinCalderón De Anda, FroylanMurase, SachikoCalderón De La Barca, Ana MaríaMurata, SoichiroCalì, TitoMurata, TakayukiCaligo, Maria AdelaideMuratori, MonicaCalina, DanielaMurcha, MonikaCałka, JarosławMurdaca, GiuseppeCallaini, GiulianoMurga, CristinaCalum, WilsonMuriel, PabloCalvisi, Diego FrancescoMurooka, ThomasCalvo, Ana CristinaMurphy, BrianCalvo-Rodriguez, MariaMurphy, RonanCalzia, EnricoMurphy, William J.Câmara, Niels Olsen SaraivaMurray Stewart, TracyCamell, ChristinaMurray, RachaelCameron, DonaldMusani, VesnaCamins, AntoniMußbacher, MarionCammas-Marion, SandrineMussolin, LaraCamougrand, NadineMustafa, GhazalaCampanale, Joseph P.Mustonen, Anne-MariCampanella, ClaudiaMus-Veteau, IsabelleCampbell, DouglasMuszyński, SiemowitCampbell, Grant R.Muthuramu, IlayarajaCampbell, KirkMuthusamy, NagendranCampello, ElenaMutis, TunaCampione, ElenaMutoh, TatsuroCampo-Engelstein, LisaMutsaers, Steven E.Campos, ReinaldoMuxel, Sandra MarciaCampos, Samuel K.Muzza, MarinaCamps, JordiMyers, Linda K.Canals, FrancescMyöhänen, Timo T.Canals, IsaacMysliveček, JaromírCanapa, AdrianaMytych, JenniferCanbay, AliNa, SungsooCancemi, PatriziaNadezhdina, ElenaCandiani, SimonaNagai, TakashiCanepari, MonicaNagano, TatsuyaCaniggia, IsabellaNagaoka, SatoshiCanipari, RitaNagaraj, ChandranCao, ChuanhaiNagarajan, Uma M.Cao, HongcuiNagata, NaotoCao, JingliNagata, YosukeCao, LingNagatomo, YujiCao, RenzhiNagayama, YujiCapel, FredericNagel, StefanCapilla, EncarnaciónNagre, NagarajaCapilla-González, VivianNagy, BálintCapitanio, NazzarenoNagy, NadineCappelletti, VeraNagy, NandorCappellini, FrancescaNagy, TamásCapuano, CristinaNahum, AmitCaraglia, MicheleNaillat, FlorenceCarazo, AlejandroNaim, ValeriaCarberry, PatrickNair, GopikaCarboni, LuciaNair, Meera G.Cardona, FernandoNajimi, MustaphaCardone, Maria FrancescaNakada-Tsuku, KumikoCardoso, Bruno AntónioNakagawa, TakahikoCardoso, Danon ClemesNakagawa, TakuroCardoso, SusanaNakagomi, TakayukiCarducci, FedericaNakahara, TomomiCarelli, StephanaNakai, AkiraCaretta, AntonioNakajima, KeiCaretti, AnnaNakajima, KoichiCarlin, Cathleen R.Nakajima, MasatoshiCarlisi, DanielaNakamura, HiroyukiCarlos, EnrichNakamura, NobuhiroCarlton, JeremyNakamura, YoshiyukiCarluccio, Maria AnnunziataNakano, TakanariCarman, Christopher V.Nakao, LiaCarmela, NardelliNakase, HiroshiCarmen, MejíaNakata, MasanoriCarmo, AlexandreNakayama, JunCarmona, JUNakayama, Jun-ichiCarmona-Ribeiro, Ana M.Nakayama, KohCarnero, AmancioNakazawa, TsutomuCarnesecchi, JulieNanako, KawaguchiCarnevale Neto, FaustoNanba, EijiCarney, RandyNanbo, AsukaCaron, KathleenNanduri, RavikanthCaroppo, EttoreNanoff, ChristianCarrasco, ElisaNaranjo, TomásCarrer, AlessandroNarayanan, AnandCarrera, PilarNardacci, RobertaCarreras, JoaquimNarducci, Maria GraziaCarriero, Maria VincenzaNarendra, DerekCarrión, MarNarita, IchieiCarro, EvaNarváez, ManuelCarroll, James A.Nash, KevinCarroll, Lara S.Nass, NorbertCarter, WayneNatale, GianfrancoCartland, SianNatale, PaoloCaruso Bavisotto, CelesteNatalicchio, AnnalisaCaruso, ArnaldoNatarajan, SivaramanCaruso, GiuseppeNatile, GiovanniCarvalho Pimenta, DanielNault, RanceCarvalho, PedroNausch, MonikaCasadei, LuciaNava, SaraCasado-Díaz, AntonioNavarro, EstanisCasalongué, Claudia AnahíNavarro, Juan AntonioCasamassimi, AmeliaNavarro-Tito, NapoleónCasanova, MagaliNawaz, MuhammadCasar, BertaNawrath, ChristianeCasas, FrançoisNawrot, BarbaraCasasnovas, Jose MaríaNayak, Nihar Ranjan Casati, SilvanaNaylor, KariCashman, NeilNazimek, KatarzynaCashman, TimothyNaziruddin, BashooCasimiro, SandraNeddens, JoergCasini, GiovanniNedoluzhko, Artem ValerievichCassina, ValeriaNees, MatthiasCassinelli, GiulianaNegroiu, GabrielaCastagnola, MassimoNejman-Faleńczyk, BożenaCastaing, BertrandNelson, ErikCastaldo, GiuseppeNelson, Leonard JCastany, SilviaNelson, Richard W.Castellano, Joan JosepNěmcová, LucieCastelli, VanessaNemoto, KiyomitsuCastello, AngeloNervi, ClaraCastillo, KarenNesmelov, Yuri E.Castoldi, AngelaNess, ScottCastorina, AlessandroNestor, CaseyCastriconi, RobertaNettles, KendallCastro, António GilNeuhaus, WinfriedCastro, Fidel OvidioNeuhäuser, BenjaminCastro, Mariana S.Neuman, BenjaminCastro-Torres, Rubén DaríoNeumann, ArianeCastrucci, Ana MariaNeumann, BrentCatalan, MarceloNeumann, JoachimCatalani, ElisabettaNevi, LorenzoCatalin, BogdanNewsome, Timothy P.Cato, Andrew C. B.Newton, Jason CCaton, PaulNeyns, BartCauli, OmarNg, RayCava, ClaudiaNg, Ray KitCavallaro, SebastianoNgezahayo, AnacletCave, John W.Nghe, PhilippeCayre, MyriamNguyen, QuynhCebecauer, MarekNguyen, TinCebrián, RubenNguyen-Tu, Marie SophieCeccarelli, SimonaNi, XiaoCelec, PeterNicaise, CharlesCelli, AnnaNicita, FrancescoCenni, VittoriaNickerson, John M.Centonze, DiegoNiclou, SimoneCeranowicz, PiotrNicola, Juan PabloCerbo, Alessandro DiNicole, SophieCerdà, JoanNicolini, GiuseppinaCermak, LukasNicosia, AldoČerná, MarieNiculita-Hirzel, HélèneCha, Hwa JunNie, ShuyiChaaban, HalaNie, YuChaber, RadosławNielsen, Vance GirardChacko, Jenu VargheseNiessing, DierkChagraoui, AbdeslamNifli, Artemissia-PhoebeChai, JinNighot, PrashantChakrabarti, KausikNikolaidis, NikolasChakrabarti, RajarshiNikolaidis, Pantelis T.Chalkias, AthanasiosNikolakaki, EleniChalla, DilipNikolakopoulou, Angeliki M.Chambers, Joseph ENiland, StephanChamcheu, Jean ChristopherNilsson, GunnarChami, MouniaNing, LiqunChan, AndrewNing, ShunbinChan, ClementNirala, Niraj K.Chan, Koon-HoNishi, RyotaroChan, Sunny S.K.Nishibori, MasahiroChan, Vera Sau-FongNishigaki, TakuyaChan, WaikinNishikawa, MasakiChan, Yun-ShenNishikawa, MasatoshiChandrasekar, IndraNishikimi, AkihikoChang, Ching-ChihNishimura, KenChang, Ching-JinNivedita, NiveditaChang, Chuang-RungNiyibizi, ChristopherChang, EunjuNizhnikov, Anton A.Chang, Heng-YuNjar, Vincent C.O.Chang, HyunNjus, DavidChang, Ing-FengNobile-Orazio, EduardoChang, JiangNóbrega, ClévioChang, Kuo-HsuanNobuhiko, OHNOChang, Ling-ChuNoda, MakotoChang, Tzu-ChingNoda, TakeshiChang, Wen-WeiNofer, Jerzy-RochChang, Yuan-YenNoghero, AlessioChao, Chia-TerNoguchi, SatoruChapel, AlainNoguera, Nélida InésChapuy-Regaud, SabineNoh, Ji-yoonCharriaut-Marlangue, ChristianeNollert, Matthias U.Charwat, VerenaNones, KatiaChasserot-Golaz, SylvetteNoonepalle, SatishChatterjee, IshitaNordgren, Tara M.Chatterjee, MadhumitaNordin, JoelChattopadhyay, SaurabhNoren Hooten, NicoleChaudhary, PankajNorman, KennethChaudhri, VirendraNormanno, NicolaChaurasia, BhagirathNørregaard, RikkeChavez-Calvillo, GabrielaNorris, JamesChecler, FredericNorris, PhilipChen, BaoyuNorton, CharlesChen, BinNorton, NadineChen, ChaoNorton, RayChen, ChiNosi, DanieleChen, Chien-LiangNotas, GeorgeChen, Chu-HuangNovák, JanChen, Chung-HwanNovoplansky, ArielChen, Chung-MingNowak, KrzysztofChen, Chun-LiangNowak-Sliwinska, PatrycjaChen, DavidNowikovsky, KarinChen, GuijieNuñez, RosarioChen, GuoxunNurzynska, DariaChen, HansenNuss, DonaldChen, HuNuytemans, KarenChen, HuiNyman, DagChen, JianxiongNyström, AlexanderChen, Ji-YihObaid, GirgisChen, JunjieObal, DetlefChen, Kuan-FuObeng, SamuelChen, Kuan-HsingOberdoerffer, PhilippChen, LiObermayer-Pietsch, BarbaraChen, Li-ChingObermeier, BirgitChen, LinObšil, TomášChen, Miao-HsuehObukhov, Alexander G.Chen, QianO’Callaghan, JamesChen, Qiao-HongOcampo, MarisolChen, Shuen-EiOccelli, Laurence M.Chen, Suet NeeOchiai, DaigoChen, Ta-ChingOchoa-Reparaz, JavierChen, TaipingO’Connell, Kevin F.Chen, YangO’Connor, José EnriqueChen, Ye-guangOczkowicz, MariaChen, YiliangOdenthal, MargareteChen, YTOdermatt, Pascal D.Chen, Yu-ChihOdierna, GaetanoChen, Yun-JuOdintsova, N. A.Chen, ZhihangO’Donnell, John CharlesChen, ZhongOe, YujiChénais, BenoitOffermann, AnneCheng, DeOgawa, TakehikoCheng, Hung-ChiOgi, KazuhiroCheng, Juei-TangOgórek, RafałCheng, Min-ChihO’Hara, StevenCheng, Shih-PingOhashi, KentaroCheng, XiOhishi, TomokazuCheng, XiangOhmido, NobukoCheng, Yuen YeeOhneda, KinukoChenghua, GuOhsugi, MihoCheon, Dong-JooOhtaka-Maruyama, ChiakiCheon, Yong-PilOhya, SusumuChern, Chi-LiangOikonomou, EvangelosChern, EdwardOjima, KoichiCherng, Juin-HongOkada, HideshiChernoff, JonathanOkada, ShigeruCheshenko, NataliaOkae, HiroakiChester, Adrian HOkajima, TetsuyaCheung, KarenOkamoto, HirokazuCheung, MartinOkamoto, TakayukiCheungpasitporn, WisitOkayasu, IsaoChe-Yen Wang, JosephOkazaki, YasumasaCheynier, RemiOkbay, AysuChhabra, GaganOkino, NozomuChi, Celestine N.Okoli, ArinzeChi, Richard J.Oksenych, ValentynChiabrando, DeborahOkumura, NobuakiChiabrando, Gustavo AlbertoOkuno, ScottChiang, Hao-SenOlabisi, RonkeChiang, Hsiu-MeiOláh, AttilaChiang, WeichungOláh, JuditChiaradonna, FerdinandoOlah, MartaChiaraluce, RobertaOłdak, MonikaChiba, KazuyoshiOlejniczak, MartaChicco, AdamOliani, SoniaChien, Chih-YenOliart-Ros, Rosa MaríaChim, Shek ManOliva, Anthony A.Chinen, Ludmilla Thomé DomingosOLive, DanielChini, Eduardo N.Oliveira, PatríciaChiocca, SusannaOlivier, ElodieChiocchetti, AnnalisaOliviero, FrancescaChiou, Jian-GengOllero, MarioChira, SergiuOlmo, EttoreChiu, Ing-MingOlsen, Christopher M.Chiu, Norman H. L.Olsen, JørgenChluba, JohannaOlson, Bradley J.S.C.Cho, KinsangOlson, Thomas L.Cho, Somi KimOmar, Syed HarisCho, Ssang-GooOng, Derrick Sek TongChobot, VladimírOng, QunxiangChoi, ChanghoonOngkasuwan, JulinaChoi, Cheol YongOniszczuk, AnnaChoi, DonghoOno, HirakuChoi, DonghoonOnorati, MarcoChoi, Jane RuOnori, PaoloChoi, Se-YoungOnyeagucha, Benjamin ChidiChoi, Soo-KyoungOoi, JoshuaChoi, Youkyung H.Opiela, JolantaChojnowski, AlexandreOponowicz, AgnieszkaChong, Yong PilOppedisano, FrancescaChou, Lan-SzuOpstad, Trine B.Chou, Tsung-HanO’Reilly, StevenChou, Yu-ChingOrfanos, Stylianos E.Chowdhury, FarhanOriá, Reinaldo B.Chowdhury, NityanandaOrii, Kenji E.Christ, BrunoOrinska, ZaneChrist, TorstenOrlacchio, AntonioChristians, ElisabethOrlandi, ChiaraChristina, Barja-FidalgoOrloff, MohammedChristopoulos, PetrosOrnitz, DavidChristov, ChristoOrtega, ÁngelesChristy, BarbaraOrtega-Gutierrez, SilviaChu, XiangpingOrzan, FrancescaChua, John Jia EnOrzechowski, ArkadiuszChuang, Kai-HsiangOsaki, MitsuhikoChuang, Tsung-HsienO’Shaughnessy, RyanChubarev, Vladimir N.Osmanagic-Myers, SelmaChubb, JonathanOsna, NataliaChueh, Pin JuOstersetzer-Biran, OrenChuffa, Luiz GustavoOstrovsky, OlgaChulanov, VladmirOstuni, MarianoChun, Jong TaiO’Sullivan, JamieChung, DonghoonOswald, FranzChung, SeokOsyczka, Anna MariaChung, Seung WooOteri, GiacomoChuntao, ZhaoOtero, CarolinaChurchward, ColinOtsuguro, Ken-ichiChuva De Sousa Lopes, Susana MarinaOtsuki, TakemiChyuan, I-TsuOtto, MarioCiaglia, ElenaOttosson, FilipCiampi, RaffaeleOvsepian, Saak VCiccarelli, RenataOzaki, ToshinoriCicero, ArrigoOzbun, MichelleCiciliot, StefanoOzen, MehmetCicinelli, EttoreOzretić, PetarCid, Victor JPabelick, Christina M.Ciemerych-Litwinienko, AnnaPabst, Andreas MaxCieniewski-Bernard, CarolinePabst, ThomasCiereszko, IwonaPacak, ChristinaCiesielski, GrzegorzPacini, NicolaCignarella, AndreaPadanilam, BabuCignetti, AlessandroPadilla-Martínez, Itzia I.Cildir, GokhanPadler-Karavani, VeredCilloni, DanielaPaduano, FrancescoCimini, SaraPaduch, RomanCimmino, GiovanniPadula, Matthew P.Cinar, BekirPaes, Marciano VianaCiornea, Elena TodirascuPagano, AlessandraCipolloni, LuigiPagella, PierfrancescoCirillo, CarlaPagenstecher, AxelCirone, MaraPaggetti, JeromeCisneros, BulmaroPahar, BapiCitro, SimonaPajevic, Paola DivietiClarimon, JordiPajonk, FrankClark, GregPalacios-Gimenez, Octavio ManuelClarke, ChirstopherPaladino, SimonaClarke, ChristopherPalazón, AsísClarke, Lane L.Palazzo, LucaClarke, PennyPalermo, AmeliaClavenzani, PaoloPalitti, FabrizioCleary, John D.Palladini, AriannaClement, AlbrechtPallàs Lliberia, MercèClément, NathaliePallett, LauraClevenger, Charles V.Pallotta, IsabellaClop, AlexPalmerini, MariaCobellis, GildaPalmieri, ErikaCocco, LucioPalmour, RobertaCoddou, ClaudioPalomba, RobertoCoffelt, Seth B.Palomo, ValleCohen, EhudPalsamy, PeriyasamyCohen-Salmon, MartinePalsdottir, VilborgCoirault, CatherinePalumbo, PasqualeColanzi, AntoninoPalus, MartinColetti, DarioPaluszczak, JarosławColl, RebeccaPalviainen, MariColla, EmanuelaPan, Chun-HsuCollart, Martine A.Pan, FengCollas, PhilippePan, Hung-ChuanCollavin, LicioPan, JiayiCollawn, Sherry S.Pan, YoulianColl-Bonfill, NúriaPanatala, RadhakrishnanColleluori, GeorgiaPandit, AnjaliCollings, DavidPanfoli, IsabellaCollis, SpencerPang, YonggangCollyer, EileenPanizzutti, BrunaColman, RickiPannaccione, AnnaColombatti, RaffaellaPanos, Georgios D.Colomo, LluísPantoja, David SotoColuccia, MauroPanza, LuigiColumbano, AmedeoPao, Ping-ChiehCombaret, LydiePaola, BrunCombier, Jean-philippePaolini, RossellaComi, CristoforoPapa, MicheleComincini, SergioPapa, SergioConciatori, FabianaPapaccio, GianpaoloConcordet, Jean-PaulPapadaki, CharaConcu, RiccardoPapait, AndreaConde, JavierParadowska-Gorycka, AgnieszkaCondello, MariaParaskeva, EfrosyniConigliaro, AlicePardo, LuiConn, Crystal S.Parekh, AnantConnell, Sean R.Paret, ClaudiaConoci, SabrinaParfenova, HelenaConover, CherylParikesit, Arli AdityaConsolino, LorenaParimon, TanyalakConstantin, BrunoParis, JasonConstantinescu, Cris SParisi, SilviaConstantinou, IordaniaPark, Chan YoungContestabile, RobertoPark, Chang HwanConti, LauraPark, DaehoConti, LucianoPARK, Eun JeongConti, PioPark, Han-AContreras, OsvaldoPark, Hwan-WooConway, Daniel E.Park, Hye YunConway, James RWPark, Jin-SungCook, GrahamPark, Joo-CheolCooke Bailey, Jessica N.Park, Joon-haCool, Robbert H.Park, Jun HyoungCooper-Knock, JohnathanPark, Jung-hyunCopland, MhairiPark, Kwang-hyunCoppey, MathieuPark, Kyung-soonCoppini, RaffaelePark, MargaretCorbet, CyrilPark, Sang ChulCordani, MarcoPark, SerkinCordaro, MarikaPark, Simone L.Cordeiro, Ana SaraPark, SungkwonCordenonsi, MichelangeloParker, Tony JCordo Russo, Rosalia I.Parma, PietroCornejo, AlbertoParnell, EuanCornelius, Lynn AnneParola, MaurizioCorradi, AnnaParpura, VladimirCorrea-Basurto, JoséParri, RheinalltCorreia-de-Sá, PauloParrish, Nicholas F.Corrochano, SilviaParrotta, ElviraCortés-Canteli, MartaParsons, JasonCortese, KatiaPasca, SergiuCortesi, LauraPascal, LauraCorthay, AlexandrePascale, RosaCorti, ChiaraPascual, JesusCorvaglia, LuigiPasetto, AnnaCorvalan, AlejandroPashov, AnastasCosentino, Nicola M.Pasinetti, Giulio MariaCosenza, MariaPasquinelli, GianandreaCosta, BarbaraPasternak, Taras P.Costa, Blaise MathiasPastorek, JaromirCosta, JuliaPastores, Gregory M.Costa, Tiago Januário DaPatel, DevenCosta-Almeida, RaquelPatel, Girijesh KumarCostamagna, DomizianaPatel, NibeditaCostanzo, VincenzoPaterlini-Bréchot, PatriziaCosta-Pinto, Ana RitaPathak, DhrubaCostarelli, LeopoldoPathmasiri, WimalCostell, MercedesPatini, RomeoCôté, FrancinePatra, AmiyaCottrell, GraemePatrignani, PaolaCoudert, Amélie EPatruno, AntoniaCoulouarn, CédricPatruno, MarcoCoulson-Thomas, Vivien JanePatrussi, LauraCousins, FionaPatten, Shunmoogum A.Couso, InmaculadaPattnaik, AsitCoutant, FrédéricPauli, José RodrigoCoutard, BrunoPavlik, Edward J.Coutinho, Maria FranciscaPavlov, TengisCouture, RéjeanPawar, Shrikant DCouvineau, AlainPawelec, GrahamCovino, RobertoPayer, BernhardCowell, IanPayne, AnnetteCowling, RandyPeake, NicholasCox, Charles D.Pearen, MichaelCraig, MorganPearl, Michael L.Cram, Erin JPearring, JillianCrawford, LisaPears, CatherineCrezee, JohannesPearson, John F.Criado, Paulo RicardoPeart, JasonCrichton, Robert R.Pec, MartinCrinelli, RitaPecina, PetrCrinò, Stefano FrancescoPecorari, GianCarloCristóbal, IonPedersen, Per AmstrupCristofani, RiccardoPedeux, RémyCrnkovic, SlavenPedone, ElisaCrompton, TessaPedraza Chaverri, JoséCrook, Zachary R.Pejler, GunnarCroset, MartinePeláez, RafaelCrosio, ClaudiaPellegrinelli, VanessaCross, MichaelPellicano, RinaldoCross, RyanPeluso, AndreaCrow, Timothy J.Pena, BrisaCruz, Homero Reyes De LaPeng, LifengCruz, Silvia LoreniaPeng, TianqingCruz-Fuentes, Carlos S.Penning, BryanCsányi, GáborPenning, LouisCsepany, TundePenzo, MariannaCsordas, GyorgyPeperzak, VictorCuadrado, AntonioPeraldi, PascalCui, JuanPercipalle, PiergiorgioCui, TiantianPerego, CarlaCui, XiaoyingPerego, MichelaCummins, Nathan W.Perera, RushikaCurcio, AlbertoPérez Tur, JordiCurti, DanielaPerez, Vinicio A. de JesusCurtis, MarionPérez-Caballero, LauraCutone, AntimoPérez-Campo, Flor MaríaCuzzocrea, SalvatorePérez-Sánchez, CarlosCvetanovic, MarijaPérez-Santos, MartínCytlak, UrszulaPerini, PaoloCzamara, KrzysztofPerks, ClaireCzapiewski, PiotrPerl, AndrasCzystowska-Kuzmicz, MalgorzataPermyakov, EugeneCzyżewski, KrzysztofPero, Maria ElenaD Prestwich, Robin JPero, RaffaelaD’Argenio, ValeriaPerrais, DavidDa Costa Kalaza, KarinPerrella, MarkDa Silva, Anabela BernardesPerrot, TaraDaci, ArmondPerrotta, CristianaD’Acquisto, FulvioPerrotta, FabioDaga, Rafael R.Perrotti, VittoriaDagda, RubenPersano, StefanoDagda, Ruben K.Perše, MartinaD’Agnano, IgeaPerugorria, Maria JesusDagnino, AlexiesPeruzzi, FrancescaDagrosa, María AlejandraPešić, MilicaD’Aguanno, SimonaPessler, FrankDaher, João P. L.Pestell, RichardDai, Dao-FuPestov, DimitriDai, ZhiyuPeters, ChristianDakhlallah, DuaaPeters, DonnaDal Col, JessicaPeters, VerenaDalal, YaminiPeters-Golden, MarcDalan, RinkooPetit, Patrice X.Dalgaard, LouisePetiti, JessicaDamia, GiovannaPetkov, StoyanDamiani, DanielaPetreaca, Ruben CDammann, ReinhardPetrella, LisaDammermann, WernerPetrillo, SaraDamron, Timothy A.Petropoulos, IsabelleDandawate, PrasadPetros, Timothy J.Dang, WeiweiPetrosino, MariaD’Angiolella, VincenzoPfänder, StephanieDaniel, Morales CanoPfeffer, Lawrence M.Daniellou, RichardPfeffer, PeterDanilenko, VNPfisterer, SimonDanker, KerstinPflughoeft, KathrynDanti, SerenaPhillips, Jennifer B.D’Antonio, MatteoPiano, IlariaDao Thi, Viet LoanPiazza, OrnellaD’Apuzzo, FabriziaPicca, AnnaD’Arcy, PadraigPiccirillo, SaraDario, BrunettiPichler, VerenaDas, BiswadipPickering, RaeleneDas, RajdeepPicketts, David JDas, RupaliPickrell, AliciaDas, ViswanathPicó, F. XavierDasgupta, SubhamoyPiekorz, Rolan P.Dash, BirajaPierantoni, RiccardoDaubon, ThomasPierdomenico, Johnny DiDaughdrill, Gary W.Pierini, MichelaDave, SandeepPierre, SandrineD’Aversa, Teresa G.Pievani, AliceDavey, MartinPijls, BartDavid, AlexandrePileczki, ValentinaDavid, Laurent I.B.Pileri, AlessandroDavignon, Jean-lucPiliponsky, AdrianDavis, DarrylPilones, Karsten AndersonDavis, David A.Pilotto, AndreaDavis, IanPina, CristinaDavis, Richard E.Pinakoulaki, EftychiaDawes, AdrianaPinar, AnitaDay, Andrew S.Pinart, ElisabethDaza-Navarro, PaulaPindyurin, Alexey V.D’Azzo, AlessandraPineda, Jose RamonDe Almeida, AndreiaPinho, VanessaDe Almeida, Rodrigo CoutinhoPinto-Duarte, AntónioDe Amicis, FrancescaPintor Dos Reis, PatríciaDe Angelis, BarbaraPiomboni, PaolaDe Assis, Leonardo V MPiotrowska, EwaDe Bari, LidiaPiotrowska, KatarzynaDe Benedetti, ArrigoPiovan, ErichDe Bruijn, Henriette S.Pires, Liliana RDe Candia, PaolaPires, O.r.De Carvalho, Litia AlvesPirkmajer, SergejDe Castro, Gabriela S.Pisapia, PasqualeDe Curtis, IvanPiskacek, MartinDe Falco, ElenaPittalà, ValeriaDe Felice, MarioPittaluga, AnnaDe Francesco, FrancescoPittet, BrigitteDe Gonzalo-Calvo, DavidPivovarova-Ramich, OlgaDe Graaf, PetraPlant, Timothy D.De Guglielmo, GiordanoPlatta, HaraldDe Kock, JoeryPlattner, RinaDe La Herrán, RobertoPlaza-Díaz, JulioDe La Monte, SuzannePlaza-Zabala, AinhoaDe La Rosa, JorgePLENCHETTE, StéphanieDe La Vega, LaureanoPliss, Artem M.De Luca, AntonellaPłociński, PrzemysławDe Luca, PaolaPlotegher, NicolettaDe Martin, SaraPlukker, John T. M.De Masi, RobertoPo, AgneseDe Matos Simoes, RicardoPobbati, AjaybabuDe Matteis, Maria AntoniettaPochynyuk, OlehDe Melo Reis, Ricardo AugustoPodar, KlausDe Meyts, PierrePoddighe, DimitriDe Miglio, Maria RosariaPoelmann, RobertDe Miguel, DiegoPoetsch, AnsgarDe Pasquale, ValeriaPoggi, AlessandroDe Robertis, MariangelaPohl, SebastianDe Rocquigny, HuguesPohlkamp, TheresaDe Rosa, EnricaPohóczky, KrisztinaDe Rubeis, SilviaPoirier, Guy G.De Simone, AngelaPoissy, JulienDe Simone, Salvatore GiovanniPoitelon, YannickDe Souza Fonseca Guimarães, FernandoPóka, RóbertDe Vries, JanPokrovsky, VadimDe, PradipPolak, Marta EwaDealy, Caroline N.Polakowski, Nicholas J.Dean, GreggPolge, CécileDean, Mason N.Poli, AlessandroDeane, James A.Polisetti, NareshDebyser, ZegerPolit, JustynaDechant, ReinhardPollak, DanielaDechat, ThomasPolo, SophieDeep, GaganPoltronieri, PalmiroDeevska, GerganaPoma, AdolfoDegan, PaoloPoma, PaolaDegano, Irene RPompili, ElenaDegiacomi, GiuliaPompilio, GiulioDéglon, NicolePonsaerts, PeterDeGracia, Donald J.Pontarin, GiovannaDel Bufalo, DonatellaPontvianne, FrédéricDel Grosso, AmbraPooler, DarcyDel Pinto, RitaPoór, PéterDel Re, Dominic P.Pop, AncaDel Rio, GabrielPopa-Wagner, AurelDelenclos, MarionPope, SimonDelgado, Teresa CardosoPopgeorgiev, NikolayDelhanty, PatricPoplawski, TomaszDelhomme, NicolasPopper, Helmut H.Delisle, Jean-SébastienPorat, ZivDella Pepa, GiuseppePorat-Shliom, NatalieDella Porta, GiovannaPorcel, E.Della Ragione, FulvioPorcelli, Anna MariaDelle Monache, SimonaPorcheri, CristinaDellis, OlivierPorras, IgnacioDello Sbarba, PersioPorrini, VanessaDell’olmo, ElianaPorro, BenedettaDelorme-Axford, ElizabethPorro, ChiaraDelprat, BenjaminPors, KlausDemarquoy, JeanPorta, HelenaDeme, PragneyaPorto, BárbaraDemonbreun, AlexisPorunelloor, MathewDen Dunnen, JeroenPoschenrieder, CharlotteDeng, Win-PingPostle, AnthonyDeng, YanxiangPotaczek, Daniel P.Denolly, SolènePoulain, Fabienne E.Depping, ReinhardPouliot, RoxaneDerer, StefaniePoznański, JarosławDerler, IsabellaPrabhu, Antony HeroldDesaubry, LaurentPramanik, AvijitDescartes, MariaPrange, ReinhildDeshmukh, RupeshPrantera, GiorgioDeshpande, ShayuPrats, Anne-CatherineDeSilva, TaraPrchal, Josef T.Desprès, PhilippePredescu, SandaDettmer, UlfPrescott, AlanDeutsch, MelaniePresta, IvanDevarajan, PriyadharshiniPrévostel, CorinneDevaux, Marie-FrançoisePriante, GiovannaDevireddy, RamPrice, JeffDey, NandiniPrieto, JoseDhahbi, JosephPrigge, Elena-SophieDhandayuthapani, SubramanianPrince, ThomasDharmawardhane, SuranganiePrinz, ImmoDhingra, SanjivPritchard, MicheleDhondt-Cordelier, SandrinePrivette Vinnedge, LisaDhungel, BijayProchownik, Edward V.Dhungel, PragyeshProcino, GiuseppeDi Agostino, SilviaProdam, FlaviaDi Benedetto, BarbaraPRODANOVIC, RadivojeDi Blasio, LauraProfumo, ElisabettaDi Carlo, CostantinoProkesch, AndreasDi Donato, MarziaPronin, Alexander V.Di Felice, ValentinaProsperi, Jenifer R.Di Franco, SimoneProtasoni, MarinaDi Gioia, MarcoPrzybyło, MałgorzataDi Liegro, ItaliaPrzybylski, GrzegorzDi Mambro, RiccardoPrzygodzka, PatrycjaDi Micco, PierpaoloPuchkova, Ludmila V.Di Nardo, AnnaPuckelwartz, Megan JDi Paola, RosannaPujals, AnaisDi Pietro, NataliaPujol, AïdaDi Renzo, LiviaPula, BartoszDi Renzo, Maria FlaviaPulgar, VictorDi Stadio, AriannaPullen, NicholasDi Virgilio, FranciscoPulliainen, ArtoDi Zazzo, ErikaPurrucker, Jan ChristophDiamantidis, Michael D.Pusch, StefanDíaz-Martín, JuanPushchina, EvgeniyaDiaz-Meco, Maria T.Puthanveetil, PrasanthDíaz-Moreno, IrenePuthanveettil, SathyaDidion, Sean P.Puthier, DenisDiederichs, SolvigPuustinen, PietriDiefenbach, Russell J.Qasim, MuhammadDiener, Kerrilyn R.Qi, XiaoyangDierickx, PieterjanQian, WeiDietrich, AlexanderQiao, HuanyuDietrich, PeterQin, Xian-YangDietz, Robert M.Qiu, BinDileep, VishnuQiu, YunDimauro, IvanQuarck, RozennDimova, TanyaQuarleri, JorgeDinescu, SorinaQueiroz Monteiro, RobsonDING, LingwenQueralt, EthelDing, Ling-WenQuesada, ErnestoDing, NingQuintanilla, MiguelDing, ZonghuiQuintanilla, Rodrigo A.Dinischiotu, AncaQuintás, GuillermoDiomede, FrancescaQuirós, Luis M.Dion, Patrick A.Raaz, UweDiPietro, Luisa A.Rab, AndrásDissanayaka, WarunaRabbani, ShafaatDiviani, DarioRaben, DanDivoux, AdelineRacanelli, VitoDiwan, AbhinavRachek, LyudmilaDjabali, KarimaRadeghieri, AnnalisaDludla, Phiwayinkosi V.Rademaker, GillesDo Amaral, Ronaldo José Farias CorrêaRadicella, J. PabloDo, Minh TruongRadisky, EvetteDobolyi, ArpádRadošević-Stašić, BiserkaDobrindt, UlrichRaffel, SimonDOBROWOLNY, GabriellaRagavan, MukundanDogra, PranayRaggi, PaoloDoidy, JoanRagni, EnricoDokmeci, Mehmet RemziRagnini-Wilson, AntonellaDokudovskaya, SvetlanaRagusa, Michael J.Dolan, Brian P.Rahimi, FaridDolff, SebastianRahman, Masmudur M.Dolfini, DilettaRahman, SafikurDomagala-Kulawik, JoannaRahmel, TimDomanskyi, AndriiRaimondi, ClaudioDomenici, GiacomoRaimondi, LaviniaDomingo-Calap, PilarRajala, Raju V.S.Domingos, Pedro M.Rajamanickam, VigneshDomozych, David S.Rajpurohit, SurendraDon, AnthonyRakowski, TomaszDonaire, LiviaRakus, DariuszDonald, Claire L.Ramadan, KristijanDonato, LuigiRamaiahgari, Sreenivasa C.Donato, Rosario FrancescoRamírez-Bergeron, DianaDong, FengRamm, GeorgDonnadieu, EmmanuelRammes, GerhardDonners, MarjoRamnath, RainaDonnini, SandraRamos, ErivanD’Onofrio, GraziaRampanelli, ElenaD’Onofrio, MariapinaRampazzo, ElenaD’Onofrio, NunziaRampias, TheodorosDonoso, PaulinaRandall-Demllo, SarronDonzelli, ElisabettaRangachari, ManuDonze-Reiner, TeresaRanjan, AmareshDöppner, Thorsten R.Ranjan, KishuDorgan, Joanne F.Ranson, MarieD’Orléans-Juste, PédroRanzato, EliaDorner, AndreaRao, AtluriDörrie, JanRaphael, LisD’Orsi, BeatriceRapone, BiagioDorstyn, LorettaRaposo, BrunoDos Santos, João Miguel MarquesRapp, AlexanderDos Santos, Nuno RodriguesRashid, A. N. M. Mamun-Or-Dosil, MercedesRashid, Mohammad HarunDoss, Michael XavierRashidi, HassanDougan, Stephanie K.Rašin, Mladen RokoDouin-Echinard, VictorineRass, UlrichDoye, ValérieRastegar, MojganDrăgoi, Cristina ManuelaRatajewski, MarcinDragoni, SilviaRathinasabapathy, AnandharajanDrapala, AdrianRathinavelu, AppuDrasbek, Kim RyunRaudsepp, TerjeDrawert, BrianRauvala, HeikkiDrews-Botsch, CarolynRavazzolo, RobertoDrissen, RoyRavens, SarinaDrosten, MatthiasRay, RatnaDrouet, EmmanuelRay, SutapaDrucker, MartinRayalam, SrujanaD’Souza, RoshanRaymond, AndreaDu, HengRaynaud, CécileDu, KuoRaz, VeredDu, Xiao-JunRaze, DominiqueDu, XuexiangReali, EvaDu, YanshengRebane, AnaDu, YixingRebeck, G. WilliamDuan, JubaoRebelo, SandraDuan, ZhijunRecalde, SergioDuarte Campos, Daniela F.Recchia, AlessandraDuarte, DelfimReche, PedroDubińska-Magiera, MagdaReddy, Marpadga A.Dubrova, YuriReddy, SakamuriDubrovska, AnnaReddy, V. PrakashDuchi, SerenaReddy, Vivek K.Duda, MałgorzataRedina, OlgaDudas, JozsefRedowicz, Maria JolantaDuddy, WilliamReed, Kent M.Dudeck, AnneReed, May J.Dudek, KatarzynaReed, Miranda NicoleDudley, Jaquelin P.Rees, PaulDufour, AntoineReeve, AmyDuguez, StephanieReeves, MatthewDummer, ReinhardReginato, MauricioDumont, EliseRegner, AndreaDunaief, Joshua L.Rehmann, HolgerDunlop, ElaineReichle, AlbrechtDupont, AurélieReid, St PatrickDupont, GenevièveReikvam, HåkonDupont, JoëlleReiling, Jan H.Dupraz, SebastianRein, AlanDupre, AudeRein, TheoDuquette, PierreReinach, Peter S.Durand, Béatrice C.Reiner, AgnesDurand, BénédicteReinhard, JacquelineDurcan, Thomas M.Reinhardt, ChristophDurham, Heather D.Reinke, HansDuronio, Robert J.Reis, Celso A.Duryee, Michael J.Rejinold, SanojD’Uscio, Livius VRelat, JoanaDutta, ParthaRelic, BiserkaDutta, PranabanandaRemondelli, PaoloDveksler, Gabriela S.Ren, JunDvořáčková, MartinaRenaud, StephenDvorakova, Magdalena ChottovaRenaut, JennyDynarowicz-Łątka, PatrycjaRenner, UlrichDziegiel, PiotrRenshaw, Stephen A.Dziegielewska, BarbaraResch, UlrikeDzobo, KevinRescher, UrsulaEar, JasonRetamal, MauricioEarl, JulieRetta, Saverio FrancescoEaton, DouglasRety, StephaneEaton, Misty J.Reukov, VladimirEbihara, TakashiReuser, Arnold J. J.Eblimit, AidenReuven, NinaEbnet, KlausRex, ToniaEcelbarger, Carolyn A.Rey, Nicolas AdrianEchchannaoui, HakimŘezáčová, MartinaEcke, Thorsten HolgerRhee, Hyun-WooEdelblum, Karen L.Rhee, Ki-JongEdgell, David R.Rhee, KunsooEdhi, Muhammad MuzzammilRiar, Amanjot KaurEdin, Matthew L.Ribaldone, DavideEdward Lawler, SeanRibaldone, Davide GiuseppeEdwards, JoshuaRibas, VicenteEdwards, Steven W.Ribbat-Idel, JulikaEeckhoute, JérômeRibeiro, DanielaEeden, Freek VanRibeiro, FabiolaEfird, Jimmy T.Ribeiro, LauraEfrat, ShimonRiboni, LauraEftestøl, EinarRicci, VittorioEfthimiopoulos, SpirosRicciardi, Maria RosariaEftimie, RalucaRiccio, PaoloEgginton, StuartRice, AndrewEguether, ThibautRichard, StéphaneEhrlich, ElanaRichards, Carl D.Eichwald, CatherineRichards, Dylan JackEid, NabilRichardson, Sandra R.Eijkelkamp, Niels N.Riches-Suman, KirstenEikmans, MichaelRichter, Günther H.S.Eiró, NoemíRichter, KatrinEisenberg, TobiasRichter, Sara N.Eisenman, RobertRichter, WitoEitan, ErezRicordi, CamiloEkholm, FilipRideout, HardyEl Ghoch, MarwanRider, MarkEl Midaoui, AdilRieckmann, ThorstenEl Mourabit, HaquimaRieder, Leila E.El Sabban, MarwanRiessland, MarkusEl Shamieh, SaidRigalli, Juan PabloElbasiouny, SherifRigon, LauraEleftheriou, Eleftherios P.Rigoni, MichelaEl-Gewely, Mohamed RaafatRigopoulos, AngelosEl-Hachem, NehmeRimondi, ErikaEliasson, LenaRimpelová, SilvieEljaafari, AssiaRinaldi, CarmenElliott, BradleyRincón, Sonia V. DelElliott, MichaelRingli, ChristophEllis, PeterRiquelme, ErickElrod, John WRitter, AndreasElsherbiny, NehalRittner, Heike L.Eltit, FelipeRiuzzi, FrancescaEltsov, MikhailRivera, VictorEngel, NoraRivera, Víctor M.Engeland, Christine E.Rivera-Toledo, EvelynEngler, AnnaRivero, FranciscoEnguita, FranciscoRizos, DimitriosEnguita, Francisco J.Rizvi, Syed A. A.Enjeti, AnoopRizzarelli, EnricoEnko, DietmarRizzi Sanches, ElenEnomoto, HirayukiRizzi, LauraEntz-Werle, NatachaRizzo, MilenaErenpreisa, JekaterinaRizzolio, FlavioEri, S. SrivatsanRo, HyunjuErickson, DavidRobert, DavidErickson, TimothyRoberts, LeeErjavec, Gordana NedicRoberts, Stephen K.Erkeland, StefanRobin, Jérôme D.Ermakov, ArtemRobinson, CliveErnst Seemann, StefanRobson, Matthew JamesErrington-Mais, FionaRoca, XavierErson-Omay, Emine ZeynepRocchi, AnnaEscandon, MonicaRocha, Emily M.Escartin, CaroleRocha-Rodrigues, SílviaEscolà-Gil, Joan CarlesRoche, Marc De LaEskandarian, Haig AlexanderRoderburg, ChristophEskelinen, Eeva LiisaRodet, FranckEskelinen, Eeva-LiisaRodil, SandraEskiw, Christopher H.Rodrigo, LuisEspada, JesúsRodrigues, Alice C.Espadas Villanueva, IsabelRodrigues, Pedro MiguelEspinel-Ingroff, AnaRodrigues-Diez, RaulEspinosa-Diez, CristinaRodriguez, Gloria MarcelaEsposito, DominicRodriguez, ReneEsposito, FrancaRodríguez, Ricardo ReyesEsser, CharlotteRodriguez-Alvarez, JoseEsser, Jennifer S.Rodriguez-Cuenca, SergioEsteban, VanesaRodriguez-Lafrasse, ClaireEsteras, NoemíRodriguez-Menocal, LuisEsworthy, Robert StevenRodriguez-Revenga, LaiaEtienne, LEFAIRodriguez-Vargas, Jose M.Etxebeste, OierRodríguez-Vita, JuanEufemi, MargheritaRoduit, RaphaelEugenin, EliseoRoeb, ElkeEum, Seok-YongRoelen, BernardEun, Jung WooRogalska, AnetaEva, AlessandraRogers, Ian M.Evangelopoulos, MichaelRoh, SanghoEvergren, EmmaRojas, FedericoEveritt, JeffreyRokavec, MatjazEwers, DavidRoks, Anton JMExbrayat, Jean-MarieRolfe, Barbara E.Expósito, JoséRolla, SimonaEyras, EduardoRomagnoli, AlessandraEzhov, MaratRoman, MaciejEzquer, IgnacioRomanelli, Maria GraziaFabczak, HannaRomanenko, Svetlana A.Fábián, ZsoltRomano, GabrieleFabregat, IsabelRomano, MariaFabris, LindaRomanov, Georgy A.Facchin, FedericaRomero-Lorca, AliciaFaccio, GretaRomuald, MentaverriFaciotti, FedericaRonain Smith, BryanFactor-Litvak, PamRoncal, CarmenFaenza, IreneRondanino, ChristineFafián-Labora, Juan AntonioRong, Chan KuanFagnocchi, LucaRonga, DomenicoFahrenkrog, BirtheRoque, AliciaFaik, AhmedRosa, Adalberto LFailla, Cristina M.Rosa, AlessandroFaísca, PatríciaRosado, JuanFaitot, FrancoisRosaly, Correa-de-AraujoFajardo, Val AndrewRosati, AlessandraFalconer, RobertRosen, KirillFalzarano, DarrylRosenberg, Jens T.Falzone, LucaRosenmann, HannaFan, Guo-ChangRosenspire, A.J.Fan, Hueng-ChuenRosi, AntonellaFan, Shih-KangRosicka-Kaczmarek, JustynaFandrey, JoachimRösner, ThiesFang, JenniferRosse, CarineFang, MingzhuRosselli, FilippoFang, QingmingRossi, ElisabettaFang, ShengyunRossi, FrancaFang, Su-ChiungRossi, WaldemarFang, SungSoonRossignol, JulienFantini, DamianoRossner, MoritzFantini, JacquesRossowska, JoannaFaraldi, MartinaRossy, JérémieFarkas, AttilaRostoff, PawełFarkas, MichaelRostoker, GuyFaroni, AlessandroRota, PaolaFarr, Susan A.Rota, RossellaFarrell, RobertRotblat, BarakFarsky, Sandra H.p.Roth, Alejandro M.Fasano, MauroRoth, MichaelFasler, ElizavetaRothhammer, VeitFast, Loren D.Rotondi, MarioFato, RomanaRottenberg, HagaiFattet, LaurentRotter, NicoleFaustino, RandolphRouault-Pierre, KevinFechner, HenryRoubalova, KaterinaFedele, FrancescoRoudnicky, FilipFehrenbach, DanielRouhiainen, AriFeigin, MichaelRouhier, Matthew F.Feldman, CharlesRouleau, MatthieuFeldman, Ross D.Rounge, TrineFeliciani, ClaudioRouschop, Kasper M.A.Felli, Maria PiaRoussel, MikaëlFendrihan, SergiuRoux, KyleFendt, MarkusRovas, LinasFeneberg, EmilyRovnak, JoelFeng, HuiRovnyak, DavidFeng, Sheng-WeiRoyle, Nicola J.Feng, Wei-shengRoyo, FelixFeng, WenyiRozalski, MarcinFeng, YiRozanov, DmitriFenoglio, ChiaraRozman, DamjanaFenton, RobertRozza, ArianeFeofanov, Alexey V.Rual, Jean-FrançoisFerguson, BradleyRubattu, SperanzaFerguson, DavidRubben, AlbertFermani, SimonaRubelj, IvicaFernandes, HugoRubiolo, JuanFernandes, Tiago G.Rubis, BlazejFernández, Vanesa MartínRückert, FelixFernandez-Botran, RafaelRudd, Sean GFernández-Martínez, EduardoRudolf, EmilFernandez-Tejada, AlbertoRudolf, RuedigerFernandez-Vizarra, ErikaRueda, BoFerrari, EnricoRueff, JoséFerraro, DianaRuegg, JoelleFerreira, NelsonRuegg, MarkusFerreira, Paulo A.Ruelland, EricFerreira, RitaRuess, Dietrich A.Ferreira-Cerca, SebastienRuest, L. BrunoFerrer, IreneRuf, Viktoria C.Ferrini, FrancescoRuffinatti, Federico AlessandroFessler, JohannesRugolo, MichelaFesus, LaszloRui, LixinFeun, Lynn G.Ruijtenberg, SuzanFiacco, ToddRuiz Laza, RocíoFialová, AnnaRuiz Pérez, María VictoriaFiamoncini, JarleiRump, KatharinaField, Seth J.Ruoppolo, MargheritaFigueira, Ana Carolina MiglioriniRuperti, BenedettoFigueiredo, BonaldRuprich-Robert, GwenaëlFigueiredo-Pereira, MariaRurali, EricaFigueroa-Romero, ClaudiaRus, Horea G.Filardi, TizianaRusak, MalgorzataFilep, János G.Rusciano, Maria RosariaFilipek, AgnieszkaRussell, OliverFilipek, AnnaRusso, DomenicoFilippi, LucaRutzler, MichaelFindlay, Geoffrey D.Rützler, MichaelFinelli, CarmineRuvio, RaquelFinsterer, JosefRuzza, ChiaraFiolka, RetoRuzzene, MariaFionda, CinziaRyan, BrigidFior, RitaRybczyńska-Tkaczyk, KamilaFiorani, MaraRychahou, PiotrFiorito, VeronicaRychahou, Piotr GFirczuk, MalgorzataRyczek, NataliaFischer, AndreasRyffel, BernhardFischer, Dagmar-ChristianeRyszawy, DamianFisher, Kevin E.Ryter, StefanFisher, ScottRzepecki, RyszardFisher-Wellman, KelseyRzewuska, MagdalenaFishman, PninaRzymowska, JolantaFiszer, AgnieszkaSá, RosáliaFlak, Jonathan NSaada, AnnFleischman, Angela G.Sabatino, LauraFlemming, SvenSabirzhanov, BorisFletcher, Nicola F.Sabol, MajaFlo, Trude HelenSacchetti, BenedettoFlorean, CristinaSacco, ElenaFlores, IdhalizSachar, David BFlores, IgnacioSack, UlrichFloss, Doreen M.Saczonek, Agnieszka OwczarczykFogacci, FedericaSaddala, Madhu SudhanaFoisner, RolandSadkowski, TomaszFolco, Hernan DiegoSadoul, KarinFoley, Ann C.Sadowski, MartinFollin, BjarkeSaeki, KumikoFölster-Holst, ReginaSaeki, ToshiakiFomina, Alla FSafavi-Naeini, MitraFong, Loren G.Sager, Hendrik B.Fonseca-Alves, Carlos EduardoSaha, DipongkorFontemaggi, GiuliaSaha, NirmalyaFonteriz, Rosalba I.Saha, SubhrajitForini, Francesca S.Sahara, HiroekiFornai, FrancescoSahara, NaruhikoForner, LeopoldoSahoo, Pabitra KumarForni, MonicaSahu, BhubananandaFort, Patrice E.Saif, LindaFort, PhilippeSaini, ShikhaFortunel, NicolasSaint-Jeannet, Jean-pierreFossati, SilviaSaint-Pol, JulienFouda, AbdelrahmanSaito, TakashiFoulkes, Nicholas S.Saito, YukihiroFountain, Samuel J.Saitoh, KenjiFournie, Jean JacquesSakai, HiromichiFournier, AntoineSakamoto, Kathleen M.Fragomeni, GionataSakamuro, DaitokuFragoso, RitaSakata, NaoakiFraineau, SylvainSakaue, HiroshiFraley, Stephanie I.Sakkas, Lazaros I.Franca Junior, Marcondes CavalcanteSaksena, NitinFrancipane, Maria GiovannaSakuma, KunihiroFrancis, HeatherSakurai, YoshinoriFranco, LuisSakwe, AmosFranco, RafaelSala, ArturoFrandes, MirelaSala, ClaudiaFrank, LuizaSalazar, IvanFranquesa, MarcellaSalehzadeh-Yazdi, AliFranzke, BernhardSaler, MarcoFranzoni, GiuliaSalgar, ShashikumarFrasca, LoredanaSalifoglou, AthanasiosFratti, Rutilio A.Salinas, EvaFrazier, ThomasSallustio, BenedettaFreeman, MichaelSalman, MootazFreitas De Freitas, LucasSalmina, AllaFreitas, Ellen CristiniSalucci, SaraFreitas, Vanessa MoraisSaluk-Bijak, JoannaFrench, CurtisSalvadori, NicolaFrett, BrendanSalvetti, AnnaFreywald, AndrewSalvolini, EleonoraFrezza, ClaudioSalzer, IsabellaFribley, AndrewSamaja, MicheleFriedersdorff, FrankSamantaray, SupritiFriedman, Alan D.Samarakoon, RohanFriesel, RobertSambandam, VaishnaviFrigeri, AntonioSánchez, AránzazuFriis-Hansen, LennartSanchez-Alcazar, JoseFrisso, GiuliaSánchez-Bueno, FranciscoFritsche, EllenSanchez-Guijo, FerminFröhlich, EleonoreSánchez-Madrid, FranciscoFruci, DorianaSánchez-Mendoza, AliciaFruttero, Leonardo L.Sánchez-Pérez, Ana MaríaFry, Christopher S.Sancho-Bru, PauFu, Audrey QiuyanSancisi, ValentinaFu, JidongSanda, MiloslavFu, LianwuSander, VeronikaFu, Nai YangSanders, YanFu, XingSands, JeffFu, ZhengSanfeliu, CoralFuchs, MarcSangiolo, DarioFuchs, YaronSanjeet, MehariyaFuhrmann, GregorSanjeewa, Kalu Kapuge AsankaFujihara, HisakoSan-Segundo, PedroFujii, WataruSantabarbara, J.Fujii, YukiSantamaria-Martínez, AlbertFujimori, KoSanthanam, AbiramiFujita, AkikazuSantibañez, Juan F.Fujita, KaoriSantillo, MichaelFujita, YasuyukiSantinon, GiuliaFujiwara, NaotoSantoni, GiorgioFukada, So-ichiroSantorelli, Filippo M.Fukuda, NoboruSantos, JerranFukuhara, AtsunoriSantos-Gallego, CarlosFukui, HirokazuSantulli, GaetanoFukushima, TsuyoshiSanz, Miguel A.Fumagalli, MartaSapalidis, KonstantinosFumarola, ClaudiaSapozhnikov, Alexander M.Fumiaki, ItoSapudom, JiranuwatFunamizu, NaotakeSaracino, IlariaFunaro, AdaSaraiva, NunoFunel, NiccolaSaravanan, Prathab BalajiFunk, RichardSardu, CelestinoFunnell, SimonSarnowski, TomaszFürthauer, SebastianSarti, Alba ClaraFurukawa, HiroSarti, Francesca MariaFurusawa, YukihiroSartini, DavideFuruse, JunjiSasaki, NorihikoFuruya, HidekiSase, AjinkyaG. Dozmorov, MikhailSato, HiromiGaascht, FrancoisSato, MasamitsuGabel, DetlefSato-Bigbee, CarmenGabellini, ChiaraSatou, RyousukeGabr, MoustafaSattin, SaraGabrić, DraganaSattler, RitaGabriel, EmmanuelSattler, SusanneGabriel, RobertSaucier, CarolineGabrielsen, Odd StokkeSauer, TimGaczynska, MariaSaunders, ThomGad, AhmedSavatier, PierreGad, Annica K. B.Savic, VladimirGadad, ShrikanthSawai, HirofumiGadea, GillesSawamura, NaoyaGadjeva, MihaelaSaxena, SugandhaGaetani, LorenzoSaxena, VikasGagat, MaciejSayed, NazishGagliano, NicolettaSbaizero, OrfeoGagliano, TeresaScafidi, JosephGagliardi, Paolo ArmandoScafoglio, ClaudioGaida, MatthiasScaglione, SilviaGailly, PhilippeScarfì, SoniaGaines, Peter C.Scarlett, GarryGaiser, TimoScatena, RobertoGalanis, AlexisSchaaf, Gerben J.Galati, NickSchäfer, KatrinGalderisi, UmbertoSchaft, NielsGalelli, LucaSchalk, Isabelle J.Galesloot, TesselSchallmoser, KatharinaGalie, PeterSchaufele, FredGaligniana, MarioScheel, Troels Kasper HøyerGaligniana, Mario D.Scheffel, JörgGall Troselj, KoraljkaSchermer, BernhardGallay, PhilippeSchiavone, MarcoGallegos-Arreola, Martha PatriciaSchiebel, ElmarGalletta, Brian J.Schierwagen, RobertGalli, AlvaroSchiffer, MarioGallitano-Mendel, AmeliaSchiffmann, RaphaelGallo, GaetanoSchindler, Charles W.Gallo, GianlucaSchink, Kay OliverGalmiche, AntoineSchisler, JonathanGaloian, KarinaSchlaepfer, Isabel RGaluska, Sebastian P.Schleicher, Sabine B.Galuska, Sebastian PeterSchlieker, ChristianGalvagni, FedericoSchlussel, Michael MaiaGalvao, IzabelaSchlüter, Klaus-DieterGalvez-Valdivieso, GregorioSchmalhausen, E.V.Gambardella, JessicaSchmetzer, Helga MariaGambarotti, MarcoSchmidt, EdGamble, Karen L.Schmidt, ThomasGamet-Payrastre, LaurenceSchmidt-Arras, DirkGamper, ArminSchmidtke, GunterGan, Gregory N.Schmidtko, AchimGan, WenjianSchmidt-Ott, KaiGan, XiangchaoSchmitt, MichaelGanaie, SafderSchmitz, ThomasGanau, MarioSchmitz-Linneweber, ChristianGandellini, PaoloSchmolke, MircoGandhirajan, Rajesh KumarSchneckenburger, HerbertGanem, Neil J.Schneeberger, MarcGanesan, Latha P.Schneider, BjoernGanguly, AnutoshSchneider, JochenGangwani, LaxmanSchneiderman, EmetGanji, RakeshSchneider-Maunoury, SylvieGanley, IanSchnoor, MichaelGannage, MoniqueSchnorrer, FrankGantenbein, BenjaminSchöck, FriederGantt, ElizabethSchohn, HervéGao, BeiScholich, KlausGao, BoScholpp, SteffenGao, JinSchonewille, MartijnGao, PeisongSchönthal, Axel H.Gao, ShuaiSchreck, Karisa C.Gao, Zhan-GuoSchröder, AgnesGaragna, SilviaSchröder, Henrik DaaGaraigorta, UrtziSchröder, SusannGarau, GianpieroSchryvers, AnthonyGarbers, ChristophSchubert, Frank RichardGarcia, Anastacia MarieSchueler, JuliaGarcía, CustodiaSchuler, PatrickGarcía, MontserratSchulze-Tanzil, Gundula GesineGarcía, PalomaSchumacher, AnneGarcia, SamuelSchuurmans, Macé M.Garcia-Alvarez, AnaSchwalbe, Ruth A.García-Arranz, MarianoSchwalm, StephanieGarcía-Castro, JavierSchwartz, GregoryGarcia-Estañ, Luis PerezSchwartz, Lawrence M.Garcia-Gil, MercedesSchwarz, HerbertGarcia-Gonzalez, Miguel ASchwarz, PeterGarcía-Heredia, José M.Sciamanna, GiuseppeGarcin, DominiqueSciascia, SavinoGardie, BettyScicchitano, PietroGardikis, KonstantinosScimone, ConcettaGardner, Andrew F.Scioscia, CrescenzioGardner, Melissa K.Sciumè, GiuseppeGargioli, CesareSciumè, MariaritaGariglio, MarisaScotlandi, KatiaGarin, Marina I.Scott, CharlotteGarinis, GeorgeScotto D’Abusco, AnnaGarmash, Elena V.Scuteri, AriannaGarofalo, MariangelaSearles, Charles D.Garozzo, DomenicoSecondo, AgneseGarrigue, PhilippeSedic, MirelaGarrity, DeborahSedmera, DavidGarstka, Malgorzata AnnaSedykh, SergeyGarten, AntjeSee Hoe, Louise E.Gasa, RosaSeebach, Jörg D.Gascón, SergioSeeman, Mary V.Gasiūnas, GiedriusSeene, TeetGaspar, PauloSegarra-Marti, JavierGasparri, FabioSeguin, Cheryle A.Gasparski, AlexanderSehgal, LalitGasperini, SerenaSeiler, Magdalene J.Gassama-Diagne, AmaSeki, EkihiroGassen, Nils ChristianSekine, ShioriGasser, Susan MSeligmann, HerveGastaldello, StefanoSeluanov, AndreiGaston, J. S. HillSemina, Ekaterina V.Gaston, KevinSenel, MehmetGaszner, BalázsSensini, AlbertoGatti, LauraSeo, JinsooGatto, FrancescaSeo, Young JoonGaudieri, SilvanaSeo, Young-JinGaudio, EugenioSeol, WongiGautam, UmaSepúlveda, PilarGauthier, BenoitSerafini Fracassini, DonatellaGavalas, AnthonySerafino, AnnaluciaGawel, KingaSeravalle, GinoGazerani, ParisaSerganova, InnaGazouli, MariaSergeeva, OlgaGe, YubinSeroogy, Kim B.Gee, Heon YungSerra-Moreno, RuthGeiger, MargaretheSerrano, Javier LeonGempt, JensSerrano, MarioGendaszewska-Darmach, EdytaSerrano, PeterGenders, AmandaSerre-Beinier, VéroniqueGeneroso, Jaqueline Da SilvaSeruggia, DavideGenovese, TizianaServe, KintaGentile, CarmineSethi, GautamGeoffroy, Cédric G.Sethi, Manveen K. Geoffroy, Marie-ClaudeSettembre, CarmineGeorgakilas, AlexandrosSetty, BhuvanaGeorgescu, AdrianaSetzer, William N.Georgiadi, AnastasiaŠevčík, JanGeorgopoulou, UraniaSewald, XaverGeraci, FabianaSewell, Andrew K.Gerasimovskaya, EvgeniaSeya, TsukasaGerbino, AndreaSfacteria, AlessandraGerbino, ValeriaSfeatcu, Ionela RuxandraGerdol, MarcoSfondrini, LuciaGerisch, GüntherSgadò, PaolaGermana, AntoninoSgambato, AlessandroGermano, GiovanniSgodda, MalteGertsch, JürgShahbazi, Marta N.Gessi, StefaniaShahi, Shailesh K.Gewirtz, DavidShahnawaz, ShahnawazGewurz, BenjaminShahzad, BabarGhag, GauravShaikh, FarooqGhelli, Anna MariaShaked, YuvalGhesquiere, BartShakya, AnishaGhiani, CristinaShanahan, CathyGhiasi, Seyed MojtabaShankar, EswarGhidini, MicheleShankland, Stuart J.Ghiggeri, Gian MarcoShanmugam, MalaGhigna, ClaudiaShanmugam, MuruganandanGhila, LuizaShao, LijianGhinassi, BarbaraShao, LongquanGhita, MihaelaShao, Wen HaiGhoneum, MamdoohSharabi, AmirGhosh, ArnabSharif, JafarGhosh, AsishSharma, ArishyaGhosh, ChaitaliSharma, GeetanjaliGhosh, Paramita M.Sharma, NaveenGiacomelli, ChiaraSharma, RohitGiacomotto, JeanSharma, ShwetaGiampietro, LetiziaSharma, SudhaGianesello, LisaSharman, Edward H.Giannakouros, ThomasShaul, Yoav D.Giansanti, Maria GraziaShaw, PeterGiantsis, IoannisShcherbik, Natalia V.Giaretta, ElisaShen, AimeeGibellini, LaraShen, Cheng-HuangGiblin, FrankShen, HaihongGibson, K. MichaelShen, JingshiGibson, SpencerShen, KeyueGideon, Hannah PriyadarshiniShen, LimingGierlinger, NotburgaShen, SanbingGiese, Arnaud P.J.Shen, Tang-LongGilding, Edward K.Shen, WenyunGilkes, Daniele M.Sheng, YueGill, Ravinder K.Shenkman, Boris S.Gillis, EllenShenolikar, ShirishGilloteaux, JacquesSheppard, Mary N.Gil-Martín, EmilioSherman, MichaelGimble, JeffreyShestopalov, Alexander MGimple, Ryan C.Sheth, BhavwantiGiniger, Edward S.Sheu, JimGinsberg, Stephen D.Shevelyov, YuriGiordano, AntonioShi, HaifeiGiordano, AnttonioShi, YangGiordano, CarlaShi, YaweiGiordano, FlavioShiao, Young-JiGiordano, FrancescaShiba, KiyotakaGiorgio, MarcoShibata, TakumaGiovannelli, PiaShibata, TatsuoGiovannetti, ElisaShie, Ming-YouGiovannini, GiadaShieh, Jeng-JerGiovarelli, MirellaShiina, MarisaGiráldez Fernández, TeresaShimada, YasuhitoGirao, HenriqueShimada, YohtaGirard-Egrot, AgnesShimaoka, MotomuGiri, Anil K.Shimizu, TatsuyaGiri, HemantShimo, TsuyoshiGirnita, LeonardShimodaira, ShigetakaGiron-Gonzalez, Maria D.Shimoni, OlgaGiudici, FabiolaShin, Jae IlGiuseppina, BasiniShin, KichulGiussani, PaolaShinde, Arti V.Giusti, IlariaShintaku, HaruoGjörloff Wingren, AnetteShintani, YasushiGkika, DimitraShiota, ShiotaGlaser, KarinShirahata, TatsuyaGlass, Karen C.Shirakami, YoheiGlavač, DamjanShirasuna, KoumeiGlendining, KellyShirokikh, Nikolay E.Glotov, AndreyShivapurkar, NarayanGloushankova, Natalya A.Shiwarski, Daniel J.Gnanapragassam, Vinayaga SrinivasanShizu, RyotaGnanaprakasam, JayaShojaei, ShahlaGnanasundram, Sivakumar VadivelShokal, UpasanaGnani, DanielaShort, Nicholas JamesGoda, TadahiroShortland, PeterGodfraind, CatherineShu, SherryGodmann, MarenShu, XinhuaGoedegebuure, PeterShukla, RuchiGojo, SatoshiShukla, SiddharthGokhman, VladimirShukla, SurendraGoldberg, GaryShuo, QieGoldberg, Gary S.Shvadchak, VolodymyrGoldfinger, Lawrence E.Siddharth, SumitGoldman, James E.Siddharthan, VenkatramanGołembiowska, KrystynaSiddiqui, ImranGoljanek-Whysall, KatarzynaSiebert, Hans-ChristianGolubnitschaja, OlgaSieburth, DerekGolubovskaya, VitaSiegenthaler, JulieGolumbeanu, MonicaSiegers, Gabrielle M.Gomes, Bruno CostaSiemens, NikolaiGomes, E.D.Siemionow, MariaGómez De Cedrón, MartaSiessere, SelmaGomez, GuillermoSigal, MichaelGomez-Casati, Diego F.Siggins, Robert W.Gómez-Florit, ManuelSigvardsson, MikaelGómez-Graña, SergioSijens, Paul E.Gomez-Lucia, EsperanzaSikorski, AleksanderGómez-Ribelles, José LuísŠiler, BranislavGonçalves, Ana M. M.Silman, IsraelGonçalves, VâniaSilva, CatarinaGöndör, AnitaSilva, Ines Vieira DaGonfloni, StefaniaSilva, Nuno A.Gong, LiangweiSilva, Susana N.Gonzales, Cara B.Silva, Susana NunesGonzález Cappa, Stella MarisSilvennoinen, OlliGonzalez Halphen, DiegoSilvestre, Miguel AngelGonzález Mendoza, DanielSilvestris, EricaGonzález, ÁlvaroSilvia, CocoGonzalez, AntonioSima, LiviaGonzález, María EugeniaSimeone, PasqualeGonzález, MartaSimeoni, LucaGonzalez, RogelioSimian, MarinaGonzález, SegundoSimm, StefanGonzález-Espinosa, ClaudiaSimmen, ThomasGonzález-Laredo, Rubén FranciscoSimon, FelipeGonzález-Mesa, ErnestoSimon, Hans-UweGonzález-Navajas, José M.Simon, Scott I.González-Scarano, FranciscoSimon, ThorstenGonzález-Vázquez, ArlyngSimona, SivoriGood, Deborah J.Simoncini, StéphanieGoode, Bruce L.Simone, CristianoGopal, KeshavSimonelig, MartineGordeeva, OlgaSimonetti, GiorgiaGorelik, JuliaSimons, MikaelGorgoulis, VassilisSimonsen, AnneGorini Da Veiga, AnaSimpson, AnnGorio, AlfredoSimpson, Benjamin S.Gorji, Sara GhorbaniSimpson, David A. C.Gorman, AdrienneSingh, Amar M.Gorres, Kelly L.Singh, AnandGoswami, AnandSingh, AnupGoto, KatsumasaSingh, AnuragGoto, RiekoSingh, Brijesh KumarGötz, ClaudiaSingh, ChandraGoua, MarieSingh, Karun KGouilleux, FabriceSingh, PradeepGourmans, Marie-JoséSingh, Ravindra NGouveia, Alexandra M.Singh, SandeepGouws, ChrisnaSinha, DebottamGowda, ChandrikaSinha, RaunakGowher, HumairaSinharoy, PritamGozal, EvelyneSiniscalco, DarioGrabauskas, GintautasSinning, ChristophGrabowska, Anna M.Sioud, MouldyGraef, MartinSipos, KatalinGräf, RalphSiristatidis, CharalamposGraham, Charles H.Sirois, Martin G.Graham, Ernest MarshallSirolli, VittorioGraham, StewartSiska, Peter J.Gramignoli, RobertoSitia, RobertoGranado, Jose Maria GonzalezSivaev, Igor B.Granados-Principal, SergioSivagnanam, MamataGrand, RogerSivakumar, SushamaGrandemange, StéphanieSivaraj, Kishor KumarGrandjean, ValérieSkandalis, SpyrosGrangette, CorinneSkelding, Kathryn A.Granja, Pedro L.Skelly, DeanneGrant, GeorgeSkendros, PanagiotisGrasa, LauraSkerjanc, Ilona S.Grassi, ClaudioSkerra, ArneGrassi, GuidoSkordalakes, EmmanuelGrassmann, FelixSkotak, MaciejGratton, Jean-PhilippeSkripuletz, ThomasGrau, James W.Skriver, KarenGrau, Juan B.Skrzypski, MarekGrauslund, MortenSkundric, Dusanka S.Gravis, GwenaelleSlade, NedaGrayson, Dennis R.Sliwinski, TomaszGreco, ToddŚliwiński, TomaszGreen, ErinSłomińska-Wojewódzka, MonikaGreen, Victoria L.Smadja, DavidGreenlee, JustinSmahel, MichalGreenway, StevenSmall, DeenaGregorieff, AlexSmani, TarikGreiner, JohannesŠmída, MichalGreither, ThomasSminia, PeterGreotti, ElisaSmith Callahan, Laura A.Grewal, ThomasSmith, AnnGrewer, ChristofSmith, Jill P.Grifoni, DanielaSmith, Sinead M.Grignani, GiovanniSmits, VeroniqueGrigorov, IlijanaSmolle, Maria AnnaGrigoryan, Eleonora N.Smyth, JamesGrill, MagdalenaSoares, RaquelGrimaldi, PaolaSoares, SylviaGrimm, Marcus O.W.Soboleva, TatianaGrinberg-Bleyer, YenkelSobolevskaya, Polina A.Grinnan, Daniel C.Sobota, Radoslaw M.Groen, EwoutSobrido-Cameán, DanielGrolli, StefanoSoh, Boon SengGroschner, KlausSojka, DorothyGros-Louis, FrançoisSojka, Dorothy K.Gross, GideonSolanki, SumeetGroßhans, JörgSoldan, Samantha S.Grote, KarstenSolé Cañadas, CarlaGrove, JoeSolé, CristinaGrover, NatalieSoler, David C.Grubić Kezele, TanjaSolesio Torregrosa, Maria De La EncarnacionGruffat, HenriSolhaug, AnitaGrumati, PaoloSolich, JoannaGrundy, LukeSolinas, AntonioGrünert, UlrikeSolingapuram Sai, Kiran KumarGrünewald, ThomasSolovchenko, AlexeiGrunig, GabrieleSomarelli, JasonGrunsven, Leo VanSomasundaram, RajasekharanGrusch, MichaelSomlyai, GáborGruss, OliverSommariva, MicheleGruszczynska-Biegala, JoannaSong, BaoliangGruszka, Alicja MSong, Byeong-WookGruters, Rob A.Song, ChunhuaGrywalska, EwelinaSong, HailongGrzela, Dawid PSong, RuiGu, FengSong, ZiyiGu, JianSonnemann, JürgenGualberto, JoseSonnino, SandroGuan, FengSonntag, KaiGuan, QingdongSorge, MatteoGuan, YutingSoria, BernatGuang, ShouhongSoria, Federico N.Guardavaccaro, DanieleSorokin, VitalyGubareva, ElenaSorrell, J. MichaelGubin, DenisSosa, Rebecca A.Gudermann, TSosic, IzidorGudey, Shyam KumarSoslau, GeraldGudima, Severin O.Sossin, WayneGuerlesquin, FrancoiseSoto-Reyes, ErnestoGuerra, FloraSoto-Reyes, IleanaGuerriero, GiuliaSoulika, AthenaGuescini, MicheleSoundararajan, MeeraGuevara-Lora, IbethSounni, Nor EddineGugliandolo, EnricoSourbier, CaroleGuha, PrasunSousa, Mónica MendesGuiamet, Juan-JoséSousa, Paulo Loureiro DeGuichou, Jean FrancoisSouyri, MicheleGuidotti, AlessandroSouza, Fátima Pereira DeGuilhot, FrançoisSowers, Lawrence C.Guillaume, Thierry A.Spagnuolo, CarmelaGuillén Viejo, CarlosSpakova, TimeaGuillén, María IsabelSpampinato, SantiGuilpain, PhilippeSparagna, Genevieve C.Guimarães, FernandoSparber-Sauer, MonikaGuinamard, RomainSparling, DavidGuirado, DamiánSpassov, SashkoGuix, Francesc XavierSpee, BartGujar, Amit D.Spencer, Peter S.Gulic, TamaraSpengler, Dietmar H.Gulino, RosarioSperanza, LuisaGullberg, DonaldSperti, CosimoGulyaeva, LyudmilaSpicuglia, SalvatoreGumulec, JaromirSpiegel, MartinGundamaraju, RohitSpinelli, Francesca RomanaGuo, BaolinSpinelli, LauraGuo, ChuanyongSpitzer, DirkGuo, DandanSplichal, IgorGuo, GraceSpoerl, DavidGuo, WeiSpolaor, SimoneGuo, XiufangSpratt, Donald E.Guo, YongSpring, KevinGupta, KuldeepŠpundová, MartinaGupta, NehalSquarzoni, StefanoGupta, SanjaySquillaro, TizianaGurevich, Vsevolod V.Squires, PaulGurha, PriyatanshSridhar, SiddharthGursoy, MerviSrinivasan, RahulGustafsson, RasmusSrivastava, AkritiGutiérrez, AntoniaSrivastava, RiteshGutierrez-Aguilar, ManuelSrivastava, SiddharthGutiérrez-Juárez, RogerSrivastava, TarakGuttery, David S.Sriwastva, Mukesh KumarGuzhova, Irina V.St John, James A.Ha, PatrickStaal, Frank J.T.Ha, SanghoonStacchiotti, AlessandraHaag, FriedrichStacewicz-Sapuntzakis, MariaHaas, Stephan L.Stadlbauer, PetrHackett, Perry B.Stadnyk, AndrewHada, MegumiStagni, VenturinaHadjiargyrou, MichaelStähli, Alexandra BeatriceHadwiger, Jeffrey A.Stakos, DimitriosHadzopoulou-Cladaras, MargaritaStamper, Brendan D.Haferkamp, IlkaStanciu, Gabriela-DumitritaHafizi, SassanStange, RichardHäfner, NormanStangl, HerbertHagenbuchner, JudithStanhewicz, AnnaHagenfeld, DanielStanifer, Megan L.Hagos, Engda G.Stanisz, BeataHahne, JensStanley, RobinHailfinger, StephanStark, Lesley A.Haitao, GuoStasiolek, MariuszHajduch, EricStathopoulos, GeorgiosHajieva, ParvanaStauber, TobiasHajime, HiraseStaudinger, JeffHajjar, Katherine AmbersonStavrou, SpyridonHakami, RaminStavru, FabriziaHakkola, JukkaStawski, RobertHakovirta, Harri H.Stecca, BarbaraHalaas, ØyvindSteele, Edward J.Hałas-Wiśniewska, MartaStefan, EduardHalberg, NilsStefanini, IreneHale, MatthewStefanis, LeonidasHaley, John D.Stefanovic, BrankoHall, DuaneStefanucci, AzzurraHallberg, EinarSteffen, UlrikeHálová, IvanaSteger, GerhardHalperin, John JStehbens, SamanthaHalushka, MarcStehle, JörgHamaï, AhmedStein, ColleenHammad, Samar M.Steinbichler, TeresaHammond, Timothy G.Steinbrenner, HolgerHan, JaeseokSteinhoff, GustavHan, Jeung-WhanStella, Giulia M.Han, JinmingStenbeck, GudrunHan, Mei-LingStepanenko, Aleksei A.Han, PingpingStephens, AndrewHan, Sun-YoungStepien, EwaHanai, JunichiStevanovic, MilenaHane, MasayaStevenson-Lerner, HeatherHanisch, Franz-GeorgSteward, RobertHaniu, HisaoStewart, AdeleHannun, YusufStewart-Ornstein, JacobHansen, Axel KornerupStice, SteveHanukoglu, IsraelStinghen, Andréa Emilia MarquesHao, JiaqingStixova, LenkaHao, MingangStock, PeggyHarada, HisashiStock, WillemHarada, MamoruStockmann, ChristianHaraguchi, RyumaStojanović, SanjaHarata, MasahikoStossi, FabioHargreaves, IainStott, Jennifer B.Hariharan, SeethalakshmiStournaras, ChristosHariri, HanaaStowe, DavidHärkönen, PirkkoStradner, Martin HelmutHarrell, Carl RandallStrafella, ClaudiaHarris, Andrew R.Strano, SabrinaHarris, StevenStrappazzon, FlavieHarrison, PatrickStrasser, RichardHartke, AxelStratikos, EfstratiosHartman, MariuszStratton, Jo AnneHartmann, DanielStrid, JessicaHarvey, RobertStrisovsky, KvidoHasegawa, HiroshiStrnadel, JanHasenstein, Karl HStrohbach, AnneHashemi, MohtadinStrömblad, StaffanHashemolhosseini, SaidStruwe, WestonHashimoto, MakotoStuart, JeffreyHashimoto-Torii, KazueStudzinski, George P.Haskó, GyörgySu, RuiHata, YutakaSu, XinmingHattori, MitsuharuSu, Ying-HsiuHau, PeterSu, ZhaoqianHaubruck, PatrickSuárez, Nicolás M.Hauser, PeterSuarez-Carmona, MeggyHausser, AngelikaSubedi, LalitaHawkins, PennySubramanian, ChitraHawlitschka, AlexanderSubramaniyan, BoopathiHayasaka, HarukoSubudhi, SonuHayashi, MikioSudar, MartinaHayashi, ShinichiroSudol, MariousHayashi, TomokoSudol, MariusHayashi, YoheiSugai, TamotsuHaybaeck, JohannesSugimoto, MasayukiHazawa, MasaharuSugimoto, NaotoshiHe, GuanglongSugimura, HaruhikoHe, MeiSugiura, TsuyoshiHe, XiaochenSujobert, PierreHébert, TerrySukhorukov, VladimirHecht, MarkusSukowati, Caecilia H. C.Heczey, Andras AttilaSukumar, PiruthiviHedfalk, KristinaSuliman, Hagir BHediger, Matthias A.Sullivan, ConHegedus, BalazsSulpice, LaurentHeger, JacquelineSumazin, PavelHegyi, EszterSumida, YoshioHeideman, WarrenSummavielle, TeresaHeiduschka, PeterSun, Alfred XuyangHeinbockel, ThomasSun, Feng-HuaHeindryckx, FemkeSun, GongqinHeinonen, Krista M.Sun, Grace Y.Heitmar, RebekkaSun, HaiyanHejnova, LucieSun, JiahongHekimi, SiegfriedSun, KunfengHeldner, MirjamSun, LiHelfrich, IrisSun, Liou Y.Helgason, VignirSun, XiaodongHeller, GerwinSun, XiaofeiHellings, NielsSun, XingminHellman, LarsSun, YeHellstrand, KristofferSun, YinHelms, VolkhardSun, ZhixiongHempel, NadineSun, ZhonghuaHenderson, Michael X.Sunda, William G.Hendriks, RudiSung, Pil SooHendrix, JelleSung, Wen-WeiHenrich, DirkSung-Yon, KimHenry, Ryan A.Surapaneni, Krishna MohanHensbergen, P.J.Surendran, KameswaranHenstridge, DarrenSuso, Else Marit InderbergHeo, Jun YoungŠušor, AndrejHerling, MarcoSussman, MarkHermoso, JuanSutherland, James D.Hernández, ÁngelSutter, AndreasHernandez, JoaquimSutter, ThomasHerold-Mende, ChristelSuzuki, KeisukeHerrera, BlancaSuzuki, ShugoHerrick, Jason R.Suzuki, SusumuHerring, Bruce E.Svanvik, JoarHerrmann, Johannes M.Svirshchevskaya, ElenaHerrmann, ThomasSvitkina, TatyanaHersey, PeterSwann, KarlHershkovitz, DovSwanson, Kenneth D.Hertl, MichaelSwartz, Steven ZacharyHervouet, EricSwiatecka-Urban, AgnieszkaHeß, JochenŚwiątek, PiotrHess, Rex S.Swiatkowska, AgataHester, JoannaSydykov, AkylbekHester, MarkSykes, CÉCILEHeumann, RolfSytar, OksanaHeusch, GerdSzanto, MagdolnaHeys, JeffreySzaryńska, MagdalenaHickson, Gilles RXSzászi, KatalinHidalgo, JuanSzczepanek, JoannaHieda, MikiSzczepankiewicz, AleksandraHiggins, ClaireSzczęsny, BartoszHiguchi, AkonSzeberény, JózsefHiguchi, KeiichiSzegö, EvaHildreth, BlakeSzekeres, MáriaHildt, EberhardSzeliga, MonikaHill, StephenSzeto, Yim-TongHillion, SophieSzewczuk, Myron R.Hilliou, FrederiqueSzewczyk, Nathaniel J.Hilton, Brett J.Szigeti, KingaHimmerkus, NinaSznarkowska, AlicjaHimoto, TakashiSzteyn, KalinaHincha, DirkSztiller-Sikorska, MalgorzataHinde, ElizabethSzuhai, KarolyHinderberger, DariushSzulc-Dąbrowska, LidiaHinkel, RabeaSzyfter, KrzysztofHirabayashi, SusumuSzymanska, KatarzynaHirami, YasuhikoTabachnik Schor, Nina F.Hirata, YokoTaddei, Maria LetiziaHiratani, IchiroTae-Jin, KimHirata-Tsuchiya, ShizuTahara, HidetoshiHirbe, Angela C.Tajeddine, NicolasHiroko, Ikeshima-KataokaTakabatake, KiyofumiHirsch, EmilioTakač, IztokHirsch, IvanTakahashi, AkihisaHisanaga, Shin-ichiTakahashi, HideyukiHitomi, KiyotakaTakahashi, TetsuyaHjelmen, Carl E.Takahashi, ToshioHnia, KarimTakahashi, TsutomuHo, Cheng-MawTakai, AtsushiHo, ChengyingTAKAM KAMGA, PaulHo, Chia-cheTakamatsu, ShigeyukiHo, Han KiatTakano, KatsuraHocart, CharlesTakao, DaisukeHochrainer, KarinTakasato, MinoruHodges, CraigTakashi, SekiyaHodgkinson, ConradTakaya, TomohideHodgson, LouisTakayama, KazuoHoffmann, KatrinTakayama, KenichiHoffmann, Markus HTakeda, ShigekiHoffmann, Peter R.Takedatsu, HidetoshiHofr, CtiradTakenaga, MitsukoHogue, IanTakeshita, KyousukeHojjat-Farsangi, MohammadTakuma, KazuhiroHojo, HironoriTalati, MeghaHolaska, JamesTalora, ClaudioHolendova, BlankaTam, Yossi(Joseph)Holien, TorilTamagawa-Mineoka, RisaHolland, ThomasTamaian, RaduHolloway, Alison C.Tamaki, TetsuroHölscher, DirkTamarit, JordiHolsinger, DamainTamariz, ElisaHolt, KellyTamba, Bogdan IonelHomblé, FabriceTamura, HidetoHomma, TetsuyaTamura, TerukoHomolka, SusanneTan, Bee KHonda-Okubo, YoshikazuTan, QiHong, Jiann-RueyTan, Tuan ZeaHong, NamkoongTanabe, HirokiHong, XinTanabe, ShihoriHong, XupengTanaka, HiroakiHong, Yi-RenTanaka, HiroshiHontoria, FranciscoTanaka, KiwamuHOO, Ruby L.C.Tanaka, SatoshiHook, IngridTanaka, YoshikazuHooper, CorneliaTanaka, YukaHopkins, Benjamin D.Taneda, AkitoHopp, KatharinaTanemura, KentaroHopp, SarahTang, Bor LuenHoppstädter, JessicaTang, Chih-HsinHorai, ReikoTang, DamuHorgusluoglu, EmrinTang, JingjingHorikawa, IzumiTang, JonathanHorna, PedroTang, WalfredHorvath, AneliaTang, YaohuiHorváthová, MiraTani, MotohiroHoshi, AkihikoTaniguchi, HiroakiHoshiba, TakashiTanino, KarenHosmane, NarayanTanos, BarbaraHosokawa, NobukoTantos, ÁgnesHossain, EkramTao, FengHosseinkhani, Baharak Tao, JunyanHo-Tin-Noé, BenoîtTao, Ya-XiongHotta, RyoTapia, OlgaHouben, RolandTar, KrisztinaHoudusse, AnneTarabykin, VictorHour, Mann-JenTarallo, RobertaHoury, Walid A.Tarantino, GiovanniHouse, ImranTarasiuk, JolantaHoushdaran, SaharTarnowski, MaciejHouštěk, JosefTartey, SarangHoutman, JonTassieri, ManlioHoward, Erin W.Tasso, RobertaHoward, MartinTassone, AnnalisaHowlett, AllynTatebe, HisashiHoyer-Fender, SigridTateno, ChiseHsia, Shih-MinTatischeff, IrèneHsiao, Hui-HuaTatsumi, KoukoHsiao, Kuei-YangTattini, MassimilianoHsieh, ChengyingTatu, LaurentHsieh, Chia-HungTatullo, MarcoHsieh, Ming KunTaubert, StephenHsieh, Tsung-HanTaura, KojiroHsieh, Tusty-JuanTauriello, DanieleHsieh, Wen-TsongTausk, Francisco A.Hsieh, Yi-HsienTavanti, FrancescoHsu, Chung Y.Tavares, Luciana PHsu, Kuo-ShengTavassoly, ImanHsu, Li-SungTaves, MatthewHsu, Ren-JunTay, Ye DeeHsu, ToddTaylor, AlisonHsu, Tsung-ITaylor, Alison MarieHu, AndyTaylor, MichaelHu, DongjianTaylor, VerdonHu, GuokuTedder, Thomas F.Hu, GuoliTee, Andrew R.Hu, JianchengTegeder, IrmgardHu, JunhuiTeh, Muy-TeckHua, Kuo-FengTeif, VladimirHuang, Chi-ChangTeijeira, ÁlvaroHuang, Haw-MingTeijeiro, J. M.Huang, HuTeixeira, Fábio G.Huang, HuachaoTeixeira, Felipe KaramHuang, HuangTeixeira, José H.Huang, JijunTejero, JesúsHuang, Kuang-TzuTenenbaum, ScottHuang, TaoTeng, PengHuang, Tse-HungTeng, Yang D.Huang, Tze-SingTeng, YunHuang, Wei-ChunTeo, JeremyHuang, XiTeodoro, Jose G.Huang, XiaoTeramachi, JumpeiHuang, Ying-HsienTeramoto, YukiHuang, YuanyuTerasaki, MarkHuangfu, DanweiTerhorst, CoxHuber, RobertTerrazas, Luis I.Huber, RomanTerrinoni, AlessandroHuber, SamuelTerry, Stephen JHubert, ChrisTesařová, MarkétaHudson, NatalieTessarz, PeterHuertas Romera, María JoséTessem, Jeffery S.Hugues, StéphanieTesta, UgoHuijbers, Maartje G.Teti, GabriellaHukkanen, JanneTetta, CiroHulmi, Juha JTeves, María EugeniaHumer, ElkeThackray, AlanaHung, Ming-ShiuThaker, Youg RajHung, Shao-WenThakor, Avnesh SHung, Shih-YaThakur, Basant KumarHuntosova, VeronikaThalhammer, TheresiaHuppi, KonradThanabalu, ThirumaranHur, Jae YoungThandavarayan, Rajarajan A.Hurley, James B.Thang, SanHurwitz, ShepardThangaraj, AnnaduraiHurwitz, StephanieThangavel, ChellappagounderHutter, GregorThapa, ManojHuwiler, AndreaThaxton, JessicaHwang, Chang-ilThe, ErlindaHwang, Eun SeongTheiss, CarstenHwang, JeeyeonThekkiniath, JoseHwang, NathanielTheopold, UlrichHyland, Edel M.Théry, ManuelHytti, MariaThevananther, SundararajahHyun, Sang-HwanTheveneau, EricIaccarino, CiroThevenod, FrankIacoangeli, AlfredoThibault, GuillaumeIacono, DiegoThibault, OlivierIadarola, PaoloThiel, TeresaIadevaia, ValentinaThielemann, ChristianeIakimova, Elena T.Thodeti, CharlesIannotti, Fabio ArturoThomas, BijuIantcheva, AneliaThomas, RogerIbáñez-Costa, AlejandroThomas, SimoneIdogawa, MasashiThompson, BarryIentile, RiccardoThompson, PeterIfergan, IgalThoreson, WallaceIgea, AnaThorin-Trescases, NathalieIglesias-Bartolome, RamiroThornton, CatherineIgnjatovic, NenadThounaojam, Menaka ChanuIgotti, MariaThul, RuedigerIhrie, RebeccaThuru, XavierIjima, HiroyukiTian, FurongIkeda, MasahiroTian, JiangweiIlles, PeterTian, WeihuaIlli, BarbaraTibério, Iolanda De Fátima Lopes CalvoIlliano, PlacidoTigas, SteliosIllien-Jünger, SvenjaTikkanen, RitvaIlmarinen, TanjaTillet, EmmanuelleIm, Chang-NimTilli, Tatiana MImai, JunTimchenko, NikolaiImamoto, NaokoTimmermans, Jean-PierreImamura, MasahiroTimmons, LisaImamura, TeruhikoTimofeev, Vladimir I.Imbimbo, BrunoTinoco, Arthur D.Imperlini, EstherTiriac, HerveInga, AlbertoTirino, VirginiaIngelman-Sundberg, MagnusTiriveedhi, VenkataswarupIngley, EvanTisi, RenataInoue, JunTissier, Paul LeInoue, KazukiTitus, MarkInoue, KazushiTochigi, YukiInubushi, ToshihiroToczek, JakubIorio, EgidioToda, TakashiIorio, Marilena V.Toda, TomohisaIppolito, LuigiTodd, LaurenIraci, NunzioTodi, Sokol V.Irato, PaolaTodorova, Dessislava A.Irini, EvnouchidouTodorova, ValentinaIrles, ClaudineTodt, DanielIrrera, NatashaToellner, Kai-MichaelIrving, TomTogawa, ToruIsacson, OleToietta, GabrieleIshida, MariToillon, Robert-AlainIshiguro, HirokiTokumoto, ToshinobuIshihara, KatsuhikoTokuraku, KiyotakaIshii, KenichiroTolvanen, TuomasIshikawa, TomohisaTomaić, VjekoslavIshikawa, YasuyukiTomar, DhanendraIshimoto, TakahiroTomas, AlejandraIshizuka, TakumiTomassetti, AntonellaIsidoro, CiroTomaszkiewicz, MartaIskratsch, ThomasTomić, SimonidaIslam, JahidulTomita, HiroyukiIslam, Md SorifulTomlinson, Michael G.Islam, ShahidulTommasini, AlbertoIslam, SorifulTommasino, MassimoIsmael, SaifudeenTomoshige, ShusukeIsozaki, TakeoTomuleasa, CiprianItariu, Bianca KarlaTonazzini, IlariaIto, GentaTong, MancyIto, Ken-ichiTong, qiangIto, NaokiTong, XinItoh, SusumuTongiorgi, EnricoItou, JunjiTonks, AlexIuliano, RodolfoToorie, AnikaIvancic-Bace, IvanaTootle, Tina L.Ivanov, AndreiTopf, UlrikeIvanova, MargaritaTorgersen, Maria LyngaasIvarsson, YlvaTörnquist, KidIvey, Malina J.Torrado, EgídioIwai, AtsushiTorras, JuanIwano, TomohikoTorre, MarialuisaIwasaki, YukaTorre, OliviaIwasawa, ShunichiroTorres Aleman, IgnacioIwata, TomoyukiTorres, Adrian GabrielIwata, YujiTorres, JaumeIyer, HariniTorres, VicenteIzadifar, ZohrehTorres-Ruiz, RaulIzaguirre, GonzaloTosolini, AndrewIzawa, TetsuyaToth, RachelIzeta, AnderTotsingan, FilbertIzquierdo-Useros, NuriaTotta, PierangelaIzsvák, ZsuzsannaTouzet, PascalIzumi, KoujiTovoli, FrancescoIzzo, AngeloToyoda, MasashiJablonska, EwaTozzi, AlessandroJablonska, KarolinaTrachana, VarvaraJacenik, DamianTrajkovic, VladimirJackman, Robert W.Tramontano, DonatellaJackson, David G.Tran, PhonJackson-Weaver, OlanTranter, MichaelJacobson, GeraldineTrapani, IvanaJaconi, Marisa E.E.Trauzold, AnnaJacquin, EliseTravelli, CristinaJacquot, YvesTremblay, Jacques P.Jadavji, NafisaTremethick, DavidJaffe, LaurindaTrentin, AndreaJaganjac, MoranaTretter, LaszloJagannatha, RaoTreves, SusanJaggi, MeenaTriaca, VivianaJahani-Asl, ArezuTriantafyllou, EvangelosJaime, Diego FrancoTricarico, DomenicoJain, MamtaTrifonov, VladimirJain, ShanuTrinchera, MarcoJaiswal, DeepikaTripodi, MarcoJaiswal, JKTrippier, Paul C.Jaiswal, Jyoti K.Trivanovic, DrenkaJakas, AndrejaTroadec, Marie-BérengèreJakovac, HrvojeTroppmair, JakobJalan-Sakrikar, NidhiTrovato, MariaJamshidi, YaldaTrushina, EugeniaJanbon, GuilhemTruskey, George AJang, JichanTryfonidou, Marianna A.Jang, KyuYunTsai, Feng-ChouJang, Sung-WukTsai, JamesJanic, AnaTsai, May-JywanJanjetovic, ZoricaTsai, Pei-YinJanke, CarstenTsai, Robert Y. L.Jankowsky, Joanna L.Tsai, Wan-ChiJansen, InekeTsakmakidis, Ioannis A.Jansone, BaibaTsang, MichaelJanssen, Joseph A.M.J.L.Tschon, MatildeJanuchowski, RadosławTse, William K.F.Januschke, JensTsingotjidou, AnastasiaJao, Li-enTsinopoulos, IoannisJarosz, Daniel FTsiolaki, Paraskevi L.Jarrous, NayefTsuchiya, HiroyukiJarstfer, Michael BruceTsuda, MakotoJasińska-Stroschein, MagdalenaTsugawa, HitoshiJat, Parmjit S.Tsukahara, HirokazuJauregui, CatherineTsukamoto, SatoshiJavaheri, TaherehTsukamoto, TetsuoJaworski, TomaszTsukiyama-Kohara, KyokoJazirehi, AliTsukuba, TakayukiJaźwińska, AnnaTsunoda, IkuoJean-Quartier, ClaireTsygankov, DenisJeltsch, AlbertTsytsarev, VassiliyJendelová, PavlaTu, ZhidongJenei-Lanzl, ZsuzsaTugues Solsona, SòniaJeney, ViktoriaTulotta, ClaudiaJenkins, GarethTuosto, LorettaJenness, Mark K.Turcan, SevinJensen, Randy L.Turetta, MatteoJensen, Rebekka VibjergTurgeon, JacquesJensen, Svend BorupTuriel, MaurizioJeong, DaewonTurley, Eva A.Jeong, Hye GwangTurner, David P.Jeong, Kyung JaeTurner, Neil A.Jerebtsova, MarinaTurner, NeillJeschke, MarcTurowski, PatricJeschke, UdoTurrini, EleonoraJežová, DanielaTuru, GáborJi, PengTutino, Maria LuisaJia, DongyuTvedt, Tor HenrikJia, HongpengTwinn, DeniseJia, JunTyler, JessicaJia, KailiangTylko, GrzegorzJiang, GuochunTyszka-Czochara, MalgorzataJiang, Qiu-XingTyukmaeva, Venera I.Jiang, SizunTyurin-Kuzmin, PyotrJiang, WeiTzanakakis, George N.Jiang, YanyanTzankov, AlexandarJiang, ZiwenUbbink, MarcellusJiménez-Mateos, Eva MaríaUchida, TakahiroJimi, EijiroUeda, HiroshiJin, Hye JinUeki, ShigeharuJin, JingUgrankar, RupaliJin, Jun-OUhrig, R. GlenJin, ShaUlasov, IlyaJin, SongwanUlisse, SalvatoreJin, TengchuanUlivieri, CristinaJin, YangUllevig, SarahJin, ZhuqiuUllrich, OliverJing, RanUlmer, Bärbel M.Jirkovsky, EduardUm, SoyounJo, Eun-KyeongUmansky, SamuilJoachimiak, EwaUmapathy, GaneshJoão, CristinaUmemura, NaokiJochum, ChristophUndas, AnettaJoffre, CarineUngar, DanielJohansson, PegahUngefroren, HendrikJohansson, StaffanUnger, MarcusJohns, TerranceUntergasser, GeroldJohnson, DanielUnternaehrer, JuliJohnson, Daniel E.Unudurthi, SathyadevJohnson, Mark I.Uosaki, HidekiJohnson, NicholasUrabe, FumihikoJohnson, RajasinghUrade, ReikoJohnson, Thomas VUrata, ShuzoJohnson, Travis KaneUrbanelli, LorenaJones, Kathryn MarieUrbani, LucaJones, T. BuckyUrbano, Ana MargaridaJonsson, Anna HelenaUriarte, IkerJoo, Min WookUrra, Félix A.Jorgačevski, JernejUsai Satta, PaoloJorge, ErikaUversky, VladimirJörns, AnneUysal-Onganer, PinarJoshi, AmitUzbekov, Rustem E.Joshi, Prof. Sunil KV Ivanov, AlexanderJoshi, ShwetaV. GRAHAM, SheilaJoshita, SatoruVacca, AngeloJost, EdgarVacher, PierreJost, Wolfgang H.Vackova, IrenaJoubert, AnnieVáclavíková, RadkaJoubès, JérômeVadas, MathewJovanović, AleksandarVagnozzi, RonaldJoven, JorgeVaidotas, StankevičiusJozkowicz, AlicjaVaillant, FrancoisJóźwicki, WojciechVainonen, JuliaJozwik, AgnieszkaVaira, ValentinaJu, XinshengValable, SamuelJuan Antonio, MorenoValach, MatusJuhász, TamásValente, CarmenJulve, JosepValente, SabrinaJune, RonValenti, MariaJung, Alain C.Valenzuela, RodrigoJung, JangwookValerio, AlessandraJung, Ji HoonValis, MartinJung, Sung ChulValius, MindaugasJung, YoungmiVallat, Jean-MichelJung, Yuh-SeogValle, Luisa DallaJunier, Marie-pierreVallée, AlexandreJunker, SteffenVallender, EricJuno, JenniferValles, Soraya L.Jurk, KerstinVallet, SoniaJurkowska, HalinaValpuesta, Jose MariaJust, SteffenValsesia-Wittmann, SandrineKaambre, TuuliValtorta, SilviaKabakov, Alexander E.Valverde, Miguel ÁngelKabashi, EdorVan Bortle, KevinKabelitz, DieterVan Den Broek, DaanKachamakova-Trojanowska, NeliVan Der Sluis, Renée M.Kadenbach, BernhardVan Eys, Guillaume J.Kahlert, StefanVan Gent, DikKai, KenjiVan Grevenynghe, JulienKaigorodova, EvgeniyaVan Hook, M.J.Kaikkonen, Minna UVan Kempen, LeonKailu, YangVan Lith, SanneKain, VasundharaVan Loosdregt, JorgKaina, BerndVan Tine, Brian AndrewKainmueller, DagmarVandel, LaurenceKaiser, AnnetteVandooren, JenniferKaitsuka, TakuVanmierlo, TimKaji, KosukeVanneaux, ValérieKajta, MalgorzataVanzi, FrancescoKakinuma, SeiVáradi, CsabaKakkassery, VinodhVarani, GabrieleKakkola, LauraVaras-Godoy, ManuelKaldis, PhilippVardjan, NinaKale, AbhijitVarela-Ramirez, ArmandoKallenborn-Gerhardt, WiebkeVarga, GeorgKallifatidis, GeorgiosVarga, ZoltánKallunki, TuulaVarikuti, SanjayKaloyanova, Dora V.Varin, AudreyKaltschmidt, ChristianVasar, EeroKamada, YoshiakiVassallo, NevilleKamen, AmineVassetzky, YegorKamieniczna, MarzenaVaucher, ElvireKamiloglu, SenemVavitsas, KonstantinosKamimura, KenyaVazquez, AlexeiKamińska, IwonaVázquez, PatriciaKanada, MasamitsuVázquez-Ibar, José LuisKanakis, IoannisVe, ThomasKanazawa, MasatoVecchione, CarmineKanda, TatsuoVechetti, IvanKanda, TeruVeenman, LeoKandeel, MahmoudVeenstra, Jan AdrianusKaneda, MasahiroVega, Francisco M.Kanekiyo, TakahisaVega-Rubín-De-Celis, SilviaKaneko, GenVegeto, ElisabettaKang, CongBaoVégh, Attila GergelyKang, DongchulVelasco, IvánKang, Ellen H.Velegzhaninov, Ilya O.Kang, Kyung PyoVelentzas, Athanassios D.Kang, SeungwooVelikova, TsvetelinaKang, Sun-WoongVénéreau, EmilieKang, Tae-HongVenkatachalam, KartikKanie, OsamuVenkatadri, RajkumarKanisicak, OnurVenkatesan, Jagadeesh KKano, KiyoshiVenkatesan, ThiagarajanKant, SashiVenkateshaiah, Sathisha U.Kanugula, Anantha KoteswararaoVenteclef, NicolasKao, Shao-HsuanVentura, FrancescKapustin, AlexanderVentura-Clapier, ReneeKarabina, Sonia-AthinaVentura-Juárez, JavierKarakesisoglou, IakowosVerdaguer, EsterKaramichos, DimitriosVerdeguer, FranciscoKaranika, StylianiVerderio, ClaudiaKarantanos, TheodorosVerderio, ElisabettaKarapetis, StefanosVereb, ZoltanKarasawa, KenVergallo, AndreaKarch, JasonVergara, DanieleKardassis, DimitrisVergunst, AnnetteKarelson, MatiVerheijen, Mark H GKariya, YoshinobuVerhulst, AnjaKaroor, VijayaVerma, SureshKarpiński, Tomasz M.Verma, Suresh K.Karsak, MelihaVermeulen, Christiaan EtienneKarstedt, Silvia VonVermeulen, MarionKarwowski, BoleslawVermijlen, DavidKasai, FumioVernos, IsabelleKaschina, ElenaVeroux, MassimilianoKashaninejad, NavidVerreault, MaïtéKasprzak, AldonaVerrecchia, FranckKasprzak, Magdalena PaulinaVersacci, PaoloKassan, ModarVerset, LaurineKasten, Benjamin B.Vesa, Stefan CristianKasten-Jolly, JaneVessoni, AlexandreKasuga, KensakuVestal, Deborah J.Kasumov, TakharVetrano, Ignazio GaspareKasztan, MalgorzataVetrano, StefaniaKatalin, LumniczkyVicari, Marcelo RicardoKataoka, NaoyukiVicent Docón, María JesusKaterova, Zornitsa LandzhovaVicente, Cristina P.Kathawala, RishilVicente-Manzanares, MiguelKato, TakamitsuVictor, VictorKatsafadou, AngelikiVidal, ReneKatsila, TheodoraVidal-Puig, AntonioKatsinelos, TaxiarchisViegas, CarlosKatyal, PriyaViel, ThomasKauhanen, Susanna M.C.Viggiano, DavideKaul, MarcusVignard, JulienKaundal, RavinderVijayan, MadhuvanthiKaur, GurvinderVilaboa, NuriaKawada, KoichiVilladiego, JavierKawaguchi, NanakoVillalta, ArmandoKawai, TatsuoVilla-Morales, MaríaKawakami, ToshiVillarreal Molina, Maris TeresaKawakubo, KazumichiVillena, GrettyKAWASAKI, HiroshiVillers, AgnèsKay, Colin D.Vinall, RuthKazue, Hashimoto-ToriiVincent, BrunoKazui, ToshinobuVincent, Theresa C.Ke, Po-YuanVincenzi, FabrizioKe, YouqiangVinci, CristinaKeegan, DavidVincze, EvaKeiner, SilkeVindigni, VincenzoKejík, ZdeněkVine, Kara L.Keller, HaraldVingolo, Enzo MariaKelly, Claire M.Viola, ManuelaKelly, Gregory M.Visagie, Michelle HelenKelly, Michy P.Visani, GiuseppeKeniry, MeganViscomi, CarloKennedy, GerardVisconte, ValeriaKennedy, LindseyVishnoi, KanchanKenny, Hilary A.Vissel, BryceKepczynska, MalgorzataViswanathan, SowmyaKepinska, MartaVitale, NicolasKeresztes, AttilaVivacqua, AdeleKern, AndreasVivarelli, SilviaKerosuo, LauraVizi, E. SylvesterKesari, Kavindra KumarVladimirov, VladimirKetteler, RobinVladimirov, Vladimir I.Khambu, BilonVlahopoulos, SpirosKhameneh, Hanif JavanmardVodickova, LudmilaKhammanivong, AliVoelkers, MirkoKhan, ArshadVoellger, BenjaminKhan, HamidullahVoermans, CarlijnKhan, MohsinVogel-Mikuš, KatarinaKhan, NaeemVoigt, NielsKhan, SabbirVolarevic, VladislavKhan, Zafar K.Völgyi, BélaKhare, VineetaVolpi, ClaudiaKhedoe, Padmini S.J.Von Hoff, DanielKheradpezhouh, EhsanVon Kopylow, KathreinKhochbin, SaadiVon Schacky, ClemensKhodanovich, MarinaVon Strandmann, Elke PoggeKholodenko, IrinaVorotelyak, EkaterinaKholová, IvanaVoss, MatthiasKhoo, Bee LuanVoutsinas, Gerassimos EKhorram, OmidVozdová, MilušeKhuchua, ZazaVu, LuanKhurana, NamrataVučetić, MilicaKiang, Juliann G.Vydra, NataliaKiaris, HippokratesVygodina, Tatiana V. Kienesberger, PetraVynios, DemitriosKiesel, BarbaraWachten, DagmarKil, Eui-JoonWade, Rachael M.Kilinc, DevrimWadham, CarolKiliszek, MarekWagenmakers, AntonKilpatrick, LauraWaggoner, Stephen N.Kim Lim, Lina HsiuWagner, Kay DietrichKim, AlfredWagner, Kay-DietrichKim, Byung SooWagner, MarekKim, Cheorl-HoWagner, WolfgangKim, Dae-KwangWahli, WalterKim, DonggeonWakamiya, NobutakaKim, EllaWälchli, SébastienKim, Geon-AWali, BushraKim, HyesunWalker, Anthony L.Kim, HyoncholWalker, Douglas G.Kim, Hyun SilWaller, Edmund K.Kim, HyungsikWan, LeiKim, Hyung-SikWan, ShibiaoKim, In-BeomWang, BiaoKim, Jae-YoungWang, ChaoKim, Jong-EunWang, Chrong-ReenKim, JongminWang, ChunKim, JoungmokWang, DanKim, JunilWang, DegengKim, KijinWang, DongKim, Kyeong-ManWang, DongdongKim, Kyoung SooWang, Eddie C.Y.Kim, KyoungtaeWang, GuozhengKim, LouWang, HongbingKim, Mi NaWang, Jaw-YuanKim, Min JungWang, Ji MingKim, Sang WooWang, Jin’AnKim, Seok-JunWang, KankanKim, Seong WhoWang, LiKim, Song CheolWang, LiliKim, Tae-JungWang, LingKim, Yong-GilWang, LipingKim, Yong-SikWang, LixiaoKim, YoosikWang, Peng-HuiKim, YoungjoWang, Richard R.-CKim, Yu YeongWang, ShaobinKinfe, Thomas M.Wang, ShyiwuKing, Carolyn G.Wang, Wen-DerKipp, MarkusWang, WenjingKireev, Igor I.Wang, XianfengKirpekar, FinnWang, XinkunKirschnek, SusanneWang, XujingKirschner, KristinaWang, YanchangKishi, YusukeWang, YingKishimoto, JiroWang, Ying-HsiuKishore, RajWang, YongqiangKiskova, TereziaWang, ZhaoningKisková, TeréziaWang, ZhigaoKiss, Anna L.Wang, ZhixiangKiss, AttilaWanrooij, PaulinaKiss, PeterWard, Marie-LouiseKiss-toth, EndreWard, Roberta JKitagawa, SeiichiWardill, Hannah R.Kitchen, PhilipWardowska, AnnaKittel, AgnesWarfel, Noel AndrewKjaer, LasseWary, KishoreKlar, AgnesWarzecha, ZygmuntKlatte-Schulz, FrankaWas, HalinaKlec, ChristianeWatabe, TetsuroKlećkowska-Nawrot, JoannaWatanabe, KazuhideKlein, John R.Watanabe, KenichiroKlein, StefanieWatanabe, YasuoKleinJan, AlexWatnick, Randolph S.Kletsas, DimitrisWaugh, Mark G.Kleuser, BurkhardWautier, Jean-LucKlimczak, AleksandraWawrzyńska, AnnaKlimina, Ksenia M.Waxman, JoshuaKloc, MalgorzataWayel, JassemKlokov, DmitryWculek, Stefanie K.Klontzas, MichailWeaver, JolantaKloskowski, TomaszWebber, JasonKlusa, VijaWeber, DanielKlussmann, EnnoWeber, Daniel GilbertKluzek, StefanWeber, GeorgKnäuper, VeraWeber, JanKnepper, JaniceWeber, K. ScottKnezevic, JelenaWebster, Nicholas J.G.Knoll, MarkoWedlich-Söldner, RolandKnudsen, JakobWeger, StefanKo, Eun-juWegwitz, FlorianKobarg, JörgWei, David ChangliKobayashi, AkiraWei, DongqingKobayashi, KoichiWei, JunKobayashi, Koichi S.Wei, Jun S.Kobayashi, MakotoWei, YufengKobayashi, SatoruWeigand, Julia E.Kobayashi, TatsuyaWeinberg, JasonKobayashi, YoshiroWeinberger, FlorianKobeissy, FirasWeinhold, NielsKocgozlu, LeylaWeisel, FlorianKoch, AchimWeisman, RonitKoch-Nolte, FriedrichWeiss, MartinKocoglu, MehmetWeiss, NorbertKodidela, SunithaWeiss, Thomas S.Koduru, Srinivas V.Wellinger, RalfKoeffel, ReneWelner, RobertKoestenberger, MartinWels, WinfriedKoga, FumitakaWelsh, JaneKoga, Jun-ichiroWelsh, MichaelKögel, DonatWelting, TimKoivunen, PeppiWen, JianjunKoivunoro, HannaWen, SichengKoivusalo, Antti IlmariWen, SijinKoizume, ShiroWen, WeiKokai, Lauren EWen, YankaiKokkinakis, EmmanouilWende, WolfgangKokkola, TarjaWeng, JinshengKolaczkowska, ElzbietaWennmalm, StefanKolarik, Andrew J.Wensel, TheodoreKolarz, BogdanWenzl, RenéKolčić, IvanaWerner, Rudolf A.Koldehoff, MichaelWernersson, SaraKolesnikova, TatyanaWerry, Eryn L.Koliakos, GeorgeWessells, RobertKolosova, NataliyaWeßels, IngaKoltsova, EkaterinaWest, James D.Komada, MunekazuWesthoff, Mike-AndrewKomatsu, DavidWestmark, CaraKomel, RadovanWestra, JohannaKomosinska-Vassev, KatarzynaWetzker, ReinhardKondo, MotonariWeyd, HeikoKong, Il-KeunWeydt, PatrickKong, QingzhongWhelan, Rebecca J.Kong, WeiliWhite, FletcherKonjar, ŠpelaWichmann, ChristianKono, MasahiroWicky Collaud, ChantalKonopka, AnnaWideman, Jeremy G.Kontsevaya, IrinaWidera, DariusKoodie, LisaWidomska, JustynaKoonce, MichaelWiedemann, PhilippKopchick, JohnWiemer, Andrew JKopp, Benjamin T.Wiener, ZoltánKorac, AleksandraWienert, BeekeKorfei, MartinaWierzbicka, ElżbietaKornek, MiroslawWiesch, Julian Schulze ZurKorner, HeinrichWiest, ReinerKorolchuk, ViktorWigle, JeffreyKorolenko, Tatyana A.Wijnholds, JanKoromyslova, AnnaWiklander, OscarKorona, DagmaraWikström, MårtenKorsching, EberhardWildgruber, MoritzKorthagen, Nicoline M.Wildt, LudwigKorytowski, WitoldWiley, LukeKosan, ChristianWilkens, LudwigKoscielny, SvenWillert, KarlKoshiba-Takeuchi, KazukoWilliam, VermiKoskela, AliWilliams, Jessica A.Kosnopfel, CorinnaWillis, Dianna E.Kostareva, AnnaWillis, RohanKostrominova, TatianaWilson III, DavidKosuge, YasuhiroWilson, KatherineKothari, Vishal M.Wilting, JorgKotova, SvetlanaWilusz, Jeremy E.Kottilil, ShyamWimmer, MarkusKou, KAYAMORIWindhorst, SabineKouji, HaradaWinek, KatarzynaKoumanov, FrancoiseWiniarska-Mieczan, AnnaKouroumalis, Elias A.Winkle, MelanieKourtidis, AntonisWinkler, ChristophKoutakis, PanagiotisWinter, AlexanderKovac, StjepanaWinter, StephanKovács, Árpád FerencWistuba-Hamprecht, KilianKovacs, WernerWitalisz-Siepracka, AgnieszkaKovács-Öller, TamásWitek, Malgorzata A.Koval, AlexeyWłoga, DorotaKoval, OlgaWnuk, AgnieszkaKovalenko, Elena I.Wnuk, MaciejKovalszky, IlonaWoeller, CollynnKovanen, PetriWoetmann, AndersKowalska, KarolinaWohl, StefanieKozhevnikova, OyunaWohlleber, DirkKoziak, KatarzynaWojasiński, MichałKraft, RobertWojtkiewicz, JoannaKrajczewski, JanWölfler, AlbertKrálovec, KarelWolinski, HeimoKramvis, AnnaWollebo, Hassen S.Krasikova, Yuliya S.Wollenberg, BarbaraKrause, GünterWolnicka-Glubisz, AgnieszkaKrebs, MarkusWolozin, BenjaminKreis, Nina-NaomiWon, Kyung JongKreitschitz, AgnieszkaWong, Boon SengKremling, AndreasWong, Boon-SengKrencik, Robert C.Wong, Chi-MingKretsovali, AndronikiWong, Ching-OnKrishna, Suresh Babu NaiduWong, ChungKrishnakumar, DevadasWong, Lee LeeKrishnamoorthy, LakshmipriyaWong, Richard W.Krishnamurthy, AkilanWong, WilsonKrishnamurthy, PrasannaWong, WingKrishnan, PreethiWoo, So-YounKrishnaswamy, GuhaWoo, Sun HeeKrishner, DanielWoo, Y. JosephKristian, TiborWood, AndrewKritsiligkou, ParaskeviWoods, BenjaminKriz, JanWoodson, JesseKriz, WilhelmWoodward, JohnKrogh, NicolaiWoolbright, Benjamin L.Krokosz, AnitaWooten, MarkKróliczewska, BożenaWorch, RemigiuszKrols, MichielWosczyna, MichaelKroon, JeffreyWree, AlexanderKrueger, AndreasWree, AndreasKruger, MarlenaWrobel, TomaszKrupenko, Natalia I.Wu, Chiao-EnKruszelnicka, OlgaWu, ChuanjinKruyt, Frank A.e.Wu, ChungChunKrysko, DmitriWu, Chung-ChunKtistakis, NicholasWu, ErxiKu, Wei-ChiWu, Fu-GenKuang, ZhengWu, JianKubaczka, CarolineWu, JianjunKubicek, JanWu, KailiKubicova, LenkaWu, LiangKucharska-Mazur, JolantaWu, LindsayKuchay, Shafi M.Wu, Mei-YiKuçi, SelimWu, MingfuKudela, ErikWu, Min-HuanKudo, NobukiWu, Shu-JuKudo, TakashiWu, YanlingKudoyarova, GuzelWu, Yuan HuaKudryashova, Tatiana V.Wu, ZhihaoKuehl, MichaelWulff, HeikeKuhn, HartmutWullaert, AndyKuhn, Liisa T.Würth, RobertoKuiper, JonasXanthou, GeorginaKujawska, MałgorzataXaplanteri, PanagiotaKukley, MariaXia, YuchenKulasinghe, AruthaXiang, XinKulemann, BirteXiang, Yang KKulik, AnnaXiao, LiKulka, MariannaXiao, XiaKulkarni, AmolXiao, ZhengtaoKulkarni, PriyankaXie, JenniferKulkarni, RitwijXie, MinKumamoto, EiichiXie, QianKumar, AkhileshXie, TingKumar, AnilXie, XiaopingKumar, BinodXin, YinKumar, GauravXing, JunjiKumar, RajeshXiong, ShunbinKumar, RajivXu, ChuanmingKumar, SanjeevXu, DazhongKumar, ShaileshXu, HongyanKumar, SuneelXu, JianKumar, SushilXu, JianlinKumar, VijayXu, JinbinKumi-Diaka, JamesXu, JunwangKumsta, CarolineXu, PengfeiKung, Mei-LangXu, RongKunisada, TakahiroXu, WenyanKunz, WolframXu, XiaojiangKunzelmann, KarlXu, XinKuo, Chao-HungXu, XuehuaKuo, Cheng-ChinXu, YanKuprash, Dmitry V.Xu, Yong-jieKurakula, KondababuXue, GuangaiKurek, KyleXue, YuKurihara, ToshihideYadav, Dharmendra K.Kurniawan, Nicholas A.Yagi, HiroshiKurose, HitoshiYagihashi, SorokuKurtenbach, StefanYago, ToruKuryłowicz, AlinaYaish, MahmoudKushima, MikiYakimovich, ArturKuśnierczyk, PiotrYallapu, MuraliKusumbe, AnjaliYamada, HiroyukiKutikhin, Anton G.Yamada, TaketoKuure, SatuYamada, YosukeKuzel, Timothy M.Yamaguchi, KiyoshiKuźnicki, JacekYamaguchi, RyujiKveberg, LiseYamakuchi, MunekazuKwak, Jong HwanYamamoto, MasahiroKwak, MinseokYamamura, SoichiroKwakowsky, AndreaYamanaka, RyuyaKweon, Oh-KyeongYamasaki, MasahiroKwok, Henry Hang FaiYamashiro, SawakoKwok, Jessica C. F.Yamato, MasayukiKwon, So HeeYamauchi, AkiraKwon, Soon HyoYamawaki, HideyukiKwon, Tae-HwanYan, ChunhongLa Mendola, DiegoYan, ShengminLaBar, ThomasYan, YanLabeit, SiegfriedYan, YanlingLaboisse, Christian L.Yan, Yong-BinLabouta, Hagar IbrahimYanda, MuraliLacal, Juan CarlosYang, BaoxueLach, Michał StefanYang, Chang-HaoLacham-Kaplan, OrlyYang, Chao-YieLacina, LukasYang, Chuen-MaoLackie, Peter M.Yang, ChunzhangLacroix, BenjaminYang, GuangLadanyi, AndreaYang, JialeLadilov, YuryYang, Ju DongLadokhin, Alexey S.Yang, JunhuaLadzynski, PiotrYang, LiguoLafont, VirginieYang, LiliLafontaine, DenisYang, QienLaforenza, UmbertoYang, QiyuanLagali, PamelaYang, Shang-HsunLagane, BernardYang, Shun-FaLagarkova, MariaYang, TaoLaghezza, AntonioYang, WancaiLaguna, AriadnaYang, Weng-LangLaham-Karam, NihayYang, WentianLaher, IsmailYang, XiaofengLai, HungchengYang, YingLai, KeaneYang, Ying-YingLai, MicheleYang, Young MokLai, XinYang, YunzeLai, YanhaoYano, HajimeLai, Yu-HengYano, MasatoLakk, MónikaYao, HiroshiLakshmikuttyamma, AshakumaryYao, JianLalitkumar, Parameswaran GraceYao, JiayiLamar, John M.Yao, JunLamarche-Vane, NathalieYaron, Jordan R.Lamas, José RamónYasuda, KazukiLamatsch, DunjaYasuhara, TakaakiLamaze, ChristopheYasuhiko, KogaLamb, Bruce T.Yazawa, TakashiLamb, Laura E.Ye, FengchunLammers, MichaelYe, JinaLamm-Shalem, NoaYe, WenleiLampiasi, NadiaYen, Jui-HungLancelin, Jean-marcYeo, SynLane, Darius J. R.Yeste, MarcLanfranchi, GerolamoYew, P. ReneeLang, AlexanderYi, Chun-XiaLang, AnnemarieYi, Joo MiLang, Anthony E.Yin, LiLange, SigrunYip, Bon HamLang-Olip, IngridYip, Ping K.Lanzi, CinziaYlostalo, Joni H.Laoudj-Chenivesse, DalilaYokota, TakafumiLaoui, DamyaYokoyama, ChikakoLaPres, John J.Yoneda, ToshiyukiLapucci, AndreaYong, Kar WeyLara, LucienneYoo, Hee MinLaranjeiro, RicardoYoo, JeongsooLardelli, MichaelYoo, Jung SunLark, DanielYoo, So YoungLaRocque, JanYool, AndreaLarre, IsabelYoon, Bo-EunLarrieu, DelphineYoon, Sung-SooLarschan, EricaYoshida, AkihiroLarsen, Anders ChristianYoshida, Go J.Larson-Prior, Linda J.Yoshida, KatsunoriLarsson, OlaYoshimatsu, GumpeiLasagni, LauraYoshimura, MasamiLatacz, GniewomirYoshioka, Ken-ichiLatella, GiovanniYoshioka, KentaroLatham, MichaelYou, Hye JinLattanzi, GiovannaYou, Hyun JuLATTANZI, SIMONAYou, SeungkwonLattanzi, WandaYou, XiaonaLatunde-Dada, Gladys OluyemisiYou, ZongbingLauretani, FulvioYoung, Robert S.Lauro, ClotildeYoungstrom, Daniel W.Lauro, DavideYousefi, Behrooz H.Lauth, MatthiasYousefi, HassanLauzurica, PilarYu, Cheng-ChiaLavin, Martin F.Yu, Chia-LiLavrentieva, AntoninaYu, ChundongLawrence, MartinYu, HaiyangLawson, VictoriaYu, Han-GangLax, PedroYu, JiaLay, Chyi-HowYu, JingyouLazar, CatalinYu, JiujiuLazarev, VladimirYu, MinzhongLazarević, VladimirYu, SeongjinLazarou, MichaelYu, VictorLazo-Zbikowski, PedroYu, Wei-HsuanLazutka, JuozasYu, XiangLe Joncour, VadimYu, YanpingLe Saux, OlivierYu, YuLe Stunff, HerveYu, Yung-LuenLe Stunff, HervéYu, ZuorenLe, WeidongYuan, BingLe, Yun-zhengYuan, QuanLeabu, MirceaYuan, XuegangLeanza, LuigiYuan, ZhihongLebaron, SimonYue, John K.LeBlanc, AmandaYumura, ShigehikoLeBleu, Valerie S.Yun, Chae-OkLech, MaciejYunes, José AndrésLechel, AndreYung, Hong-WaLecona, EmilioYurchenko, Andrey A.Lecourtier, LucasYutzey, KatherineLedder, OrenYuzhalin, ArseniyLederer, Carsten WernerZaboronok, AlexanderLederkremer, Gerardo Z.Zabuchi, GiulianoLee, BethZachleder, VilemLee, Byoung DaeZachos, GeorgeLee, Chang-HoonZádori, ZoltánLee, Che-HsinZaehres, HolmLee, Dong HunZaffran, StephaneLee, Dong RyulZagórska, AgnieszkaLee, Dung-FangZagożdżon, RadosławLee, ErinnaZahid, MalihaLee, GihyunZaidi, SobiaLee, Hae-JuneZaiou, MohamedLee, Hsin-ChenZaitsev, AlekseyLee, Hsueh-TeZakharenko, Stanislav S.Lee, Hye YoungZakrzewska, MagdalenaLee, HyeminZamai, LorisLee, HyukjinZambelli, VanessaLee, JieZambrano, AngaraLee, Jong-EunZambrano, SamuelLee, Joo YoungZamperin, GianpieroLee, Jun HeeZanella, IsabellaLee, KangwonZanetti, GiuliaLee, Ki YoungZani, AugustoLee, Man RyulZannetti, AntonellaLee, OukseubZapico, Sara C.Lee, Seung HoZárate-Bladés, Carlos R.Lee, Seung HwanZarnowski, TomaszLee, Soo YoungZarobkiewicz, MichałLee, StevenZavridou, MarthaLee, SunjaeZawadzka, MalgorzataLee, Tse-MinZbigniew, BrzozkaLee, Yeon-SuŽdralević, MašaLee, Yi-JangŻebrowska, EwaLee, Yong SunZegers, Mirjam M.P.Lee, Young JinZehbe, IngeborgLee-Chang, CatalinaZeisel, Mirjam B.Lefebvre, VeroniqueZelina, PavolLefevre, Sophie D.Zella, DavideLeffler, HakonZelles, TiborLefkimmiatis, KonstantinosZeltner, NadjaLefrançois, PhilippeZemlyanskaya, Elena V.Legembre, PatrickZeng, JiaLegewie, StefanZengler, KarstenLegler, PatriciaZerges, WilliamLegouis, RenaudZhai, KuiLehane, Adele M.Zhan, XuanzhiLehmann, Brian D.ZHANG, Daniel XinLehmann, Lorenz H.Zhang, Dao QiLehotsky, JanZhang, DianbaoLei, WeiZhang, DongyunLeMaoult, JoelZhang, DuoLemonnier, LoicZhang, FanLenartowska, MartaZhang, GeLencer, WayneZhang, HuLenders, MalteZhang, HuijieLendvai, GáborZhang, JenniferLeng, TiandongZhang, JianminLennartz, MichelleZhang, JieLensen, Sarah FrancesZhang, JunLenzi, AndreaZhang, JunranLenzi, PaolaZhang, KeLéon, CatherineZhang, LinLeón, JosefaZhang, Ling-juanLeonardo, CostantinoZhang, LiqiangLeone, AngeloZhang, MingLeone, PatriziaZhang, NuLeopoldo, MarcelloZhang, PingLeow, Melvin Khee-ShingZhang, QiangLeppik, LiudmilaZhang, QiongLeru, PolianaZhang, QiuyangLesniak, WieslawaZhang, QuanLesnikowski, Zbigniew J.Zhang, RuiLeszczuk, AgataZhang, RuiwenLetek, MichalZhang, SicongLetizia, TrovatoZhang, WenboLeung, DuncanZhang, WencaiLevantini, ElenaZhang, XinxingLevaot, NoamZhang, XuexinLevi Sandri, Giovanni BattistaZhang, YixuanLévi, SabineZhang, YubinLevick, ScottZhang, YuminLevin, LONNY R.Zhang, ZhaoLevine, HerbertZhang, ZhibingLevi-Schaffer, FrancescaZhao, QingnanLevkau, BodoZhao, WeiLevy, Arkene S.A.Zhao, ZhongmingLewinski, AndrzejZheng, XiaobinLewis, AndrewZhong, ZhaohuaLeWitt, Moira S.Zhou, ChengjiLezot, FredericZhou, ChiL’homme, LaurentZhou, Heshan SamLi, BingyunZhou, JichunLi, Chia-JungZhou, JieLi, ChinZhou, JingyingLi, ChunjieZhou, LanLi, DeminZhou, MinLi, DeyaoZhou, QingyuLi, DongZhou, ShuanhuLi, FuyangZhou, YangLi, GuohuiZhou, YouwenLi, HeZhou, YunLi, HuaZhou, ZhidongLi, HuiZhou, ZhijunLi, HuinanZhu, LinLi, JiaZhu, Michael X.Li, JianrongZhu, WenjunLi, JisuZhu, WufuLi, JunZhu, WuqiangLi, Ka WanZhu, XiangdongLi, LiZhu, ZheLi, LianboZhuang, XiaohongLi, LinZhukova, NataliaLi, QingshengZiats, Nicholas P.Li, Shengwen CalvinZic, John A.Li, ShitaoZidovec Lepej, SnjezanaLi, ShuaiZieger, MarinaLi, ShuoZielezinski, AndrzejLi, SunanZielińska, SylwiaLi, TiangangZientara-Rytter, KatarzynaLi, TianhuZieseniss, AnkeLi, Tristan QingyunZigrino, PaolaLi, WeiZille, MariettaLi, Wei VivianZimmer, JacquesLI, WEIMINZimmer, SebastianLi, WenboZimmer-Bensch, GeraldineLi, Wen-hongZironi, IsabellaLi, XiangZmijewski, MichalLi, YanZmurchok, ColeLi, ZhijunŻołądek, TeresaLi, ZongliZorov, Dmitry B.Lian, QizhouZou, ChunbinLian, ZhengxingZou, HongyanLiang, ChengyuZou, JunjieLiang, JunZoumpourlis, VassilisLiang, YuyingZubiaga, AnaLiang, ZhibinZugaza, José LuisLiao, DaiqingZuloaga, Damian GabrielLiao, PengZúñiga-Hernández, JessicaLiao, Wei-BiaoZunke, FriederikeLiao, XudongZuo, LiLiao, Yi-JenZuppinger, ChristianLiao, ZehuanZuryn, StevenLiapi, CharisZwaans, Bernadette M.M.Liaw, NormanZwacka, RalfLiberton, MichelleZweigerdt, RobertLibra, MassimoZwerschke, Werner

